# Mutually exclusive pathways for biosynthesis of polyamine homospermidine dependent on putrescine or agmatine

**DOI:** 10.1016/j.jbc.2026.113510

**Published:** 2026-09-23

**Authors:** Bin Li, Hamid R. Baniasadi, Jue Liang, Margaret A. Phillips, Anthony J. Michael

**Affiliations:** Department of Biochemistry, UT Southwestern Medical Center, Dallas, Texas, USA

**Keywords:** homospermidine, agmatine, putrescine, deoxyhypusine synthase, homospermidine synthase, agmatinase, ornithine decarboxylase, arginine decarboxylase, agmatine ureohydrolase, agmatine deiminase, neofunctionalization

## Abstract

Homospermidine is a structural analog of spermidine, a polyamine that is essential for growth and cell proliferation in eukaryotes. The eukaryotic enzyme deoxyhypusine synthase (DHS) can synthesize homospermidine directly from spermidine and putrescine. Nonhomologous bacterial homospermidine synthase (HSS) synthesizes homospermidine directly from two molecules of putrescine. SpeY is a homolog of DHS in bacteria that is essential for homospermidine biosynthesis in a cyanobacterium. Recently, it was shown in the bacterial hyperthermophile *Thermus thermophilus*, that SpeY couples two molecules of agmatine produced by arginine decarboxylase to form *N*^1^,*N*^9^-*bis*(guanidino)homospermidine (G44G), which is then converted to homospermidine by a ureohydrolase. We sought to determine whether synthesis of G44G was a common feature of phylogenetically diverse SpeY homologs. Herein, we demonstrate that SpeY homologs from diverse bacterial phyla, and from species encoding arginine decarboxylases from three different protein folds, with either agmatinase or agmatine iminohydrolase partners, all form G44G. Diverse agmatine, *N*^1^-aminopropylagmatine and presumed G44G ureohydrolases convert G44G to homospermidine, but SpeY-associated G44G ureohydrolases do not act on agmatine. Similarly, diverse agmatine, *N*^1^-aminopropylagmatine and presumed G44G iminohydrolases convert G44G to *N*^1^,*N*^9^-*bis*(carbamoyl)homospermidine, but SpeY-associated G44G iminohydrolases do not act on agmatine. We identified ornithine decarboxylases in both the SpeY and HSS pathways that have become neofunctionalized to arginine decarboxylases, and in the HSS pathway are associated with an agmatinase. Bacterial homospermidine biosynthetic pathways are therefore dependent either on agmatine/SpeY or on putrescine/HSS. Our study highlights a new role for agmatine in bacterial polyamine biosynthesis, which also includes the *N*^1^-aminopropylagmatine and carboxyaminopropylagmatine pathways for spermidine production.

It is likely that almost all cells contain polyamines, small linear, flexible polycations derived from amino acids, that are fully protonated at physiological pH (1, 2). The most prominent polyamine is the triamine spermidine (Spd), which was probably present in the Last Universal Common Ancestor of all life (3). Eukaryotic cells contain Spd (Fig. 1*A*) almost exclusively as their only triamine, although some plants produce the symmetrical triamine homospermidine (Hspd) for alkaloid biosynthesis (4, 5), and some single-celled eukaryotes such as *Paramecium* species have replaced Spd with Hspd (6). In bacteria, Hspd is far more prevalent than in eukaryotes, and is the main polyamine in diverse bacterial phyla (7, 8, 9, 10).

**Figure 1:**
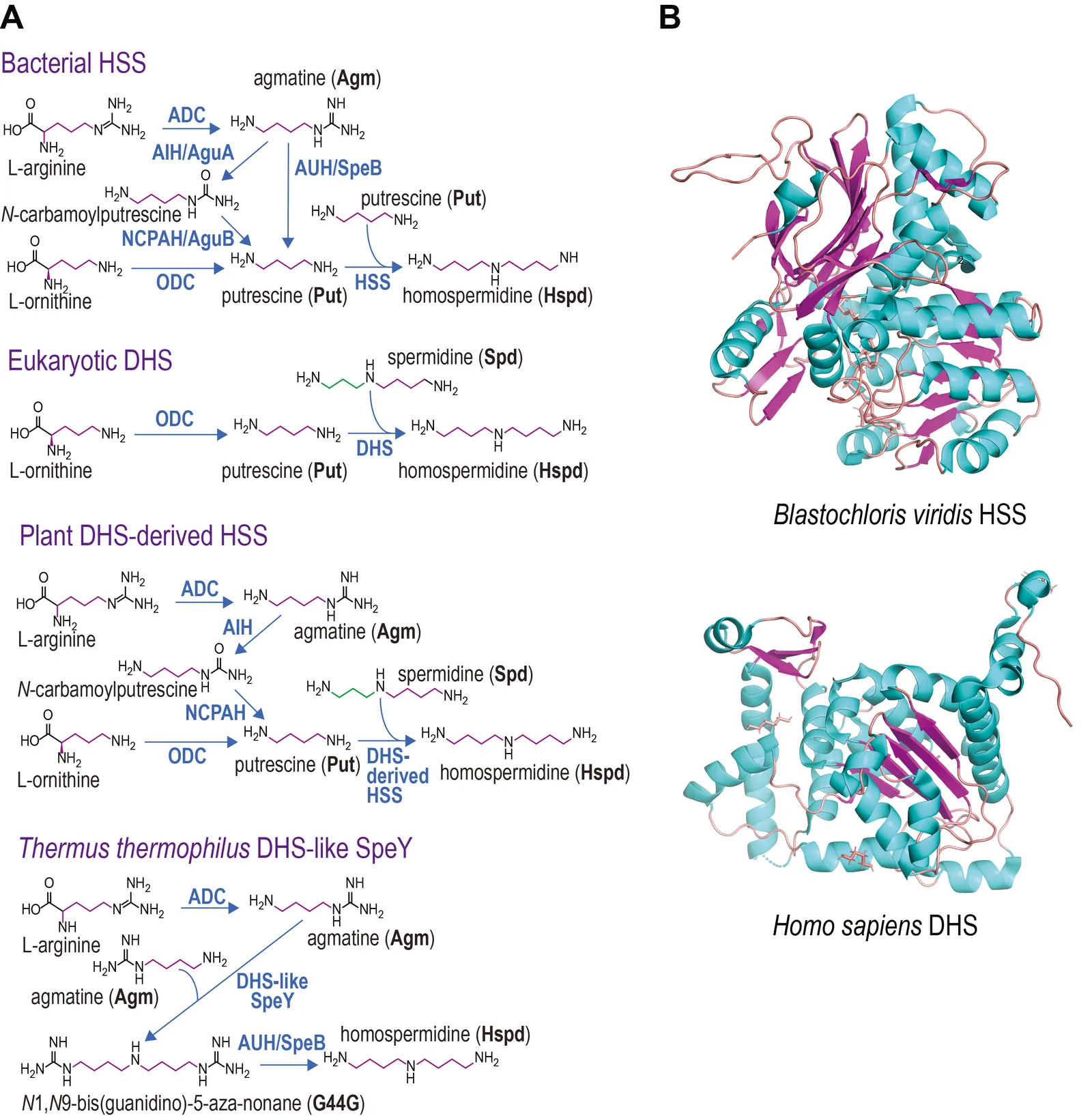
**Known enzymes and pathways for homospermidine biosynthesis.***A*, known pathways for homospermidine biosynthesis by HSS, DHS, and DHS homolog SpeY. *B*, X-ray crystal structures of bacterial homospermidine synthase (HSS) [PDB ID 4TVB] and human deoxyhypusine synthase (DHS) [6XXM].

Polyamine biosynthesis is an exemplary model for studying convergent evolution (11, 12). There are two known nonhomologous pathways for Spd biosynthesis, based on decarboxylated *S*-adenosylmethionine or aspartate β-semialdehyde. Biosynthesis of Hspd (Fig. 1*A*) is entirely different to that of Spd, and there are two known, nonhomologous biosynthetic routes (13). The first pathway was discovered in extracts of the α-proteobacterium *Rhodopseudomonas viridis* incubated with the diamine putrescine (Put) (14). This pathway consists of a single enzyme, Hspd synthase (EC 2.5.1.44; HSS), that synthesizes Hspd from two molecules of Put using NAD+ as a noncovalent cofactor (15, 16) (Fig. 1*A*). Several structures of HSS (Fig. 1*B*) are available: from γ-proteobacteria *Legionella pneumophila* (PDB ID: 2PH5) (13), *Pseudomonas aeruginosa* (17), and α-proteobacterium *Blastochloris viridis* (17, 18). The bacterial HSS (EC 2.5.1.44) possesses a highly specific site for Put binding within the active site (18) and uses another Put molecule as the aminobutyl group donor. This enzyme can also use Spd or Hspd as aminobutyl donors, but these activities are unlikely to be physiologically relevant in most bacteria. When the *B*. *viridis* HSS was incubated with agmatine (Agm), *that is*, decarboxylated arginine, although Agm was found on the surface of the HSS protein, none was detected in the active site (18). It is therefore very unlikely that Agm is a substrate for HSS. Bacterial HSS is structurally related to carboxyspermidine dehydrogenase, lysine 6-dehydrogenase, saccharopine dehydrogenase, and aspartate dehydrogenase (13, 18).

A second route for Hspd biosynthesis is evolutionarily and structurally unrelated to the bacterial HSS. In eukaryotes, the enzyme deoxyhypusine synthase (DHS; EC 2.5.1.46) transfers an aminobutyl group from Spd to a lysine residue in translation factor eIF5A to form the deoxyhypusine post-translational modification (19, 20). Hypusinated eIF5A is involved in translational initiation and elongation and is required to relieve ribosome stalling at proline-rich motifs in nascent polypeptides in the ribosome P-site (21). A side activity of DHS in the absence of eIF5A is the transfer of an aminobutyl group from Spd to Put to form Hspd (22). In some plants that produce pyrrolizidine alkaloids, DHS has been duplicated; one copy has lost its ability to bind to eIF5A and has become a Spd-dependent Hspd synthase (DHS-like HSS; EC 2.5.1.45) (5).

Bacteria also encode homologs of DHS (13) that are thought to have been acquired from an archaeal DHS by horizontal gene transfer (HGT) (23). The DHS homolog (designated as SpeY) from the filamentous, nitrogen-fixing (diazotrophic) cyanobacterium *Anabaena* sp. PCC 7120 is essential for Hspd biosynthesis and diazotrophic growth (24). It is not known by which mechanism the cyanobacterial SpeY synthesizes Hspd, however, this species does not synthesize Spd. Recently, important insight into the mechanism of Hspd biosynthesis by a SpeY homolog was described in the extreme hyperthermophile *Thermus thermophilus* (25, 26). It was found that the *T*. *thermophilus* SpeY homolog couples two molecules of Agm together to form *N*^1^,*N*^9^-*bis*(guanidino)-5-aza-nonane, *that is*, *N*^1^,*N*^9^-*bis*(guanidino)homospermidine (G44G) (Fig. 1*A*), which has been given the trivial name caldomycin. The guanidino groups of G44G are then hydrolyzed by an agmatinase/agmatine ureohydrolase (AUH/SpeB) homolog to form Hspd and urea. We sought to determine whether the mechanism of Hspd biosynthesis by SpeY homologs *via* G44G was specific to hyperthermophile *T*. *thermophilus* or was more generally distributed in bacteria. We also sought to analyze how G44G was converted to Hspd, and to identify any potential metabolic reasons for the evolution of two alternative pathways for homospermidine biosynthesis.

## Results

### Eukaryotic DHS expressed in *E*. *coli* produces homospermidine only in the presence of spermidine but SpeY produces *N*^*1*^,*N*^*9*^-*bis*(guanidino)homospermidine

To compare Hspd biosynthesis by eukaryotic DHS and bacterial DHS homologs (SpeY), we expressed the human and plant (*Arabidopsis thaliana*) *dhs* genes, and a mesophilic bacterial s*peY* homolog phylogenetically distant from *T*. *thermus*, in *Escherichia coli*. The eukaryotic DHS, in the absence of its target eIF5A, is known to produce Hspd from Put and the aminobutyl donor Spd. A *speY* homolog from the mesophilic α-proteobacterium *Candidatus Fonsibacter ubiquis*, a freshwater member of the Pelagibacterales order, was used to compare G44G and Hspd production with the eukaryotic *dhs* genes (all analyzed proteins described in Table S1). A human-type ornithine decarboxylase (ODC, alanine racemase fold) is encoded by *Ca*. *F*. *ubiquis*; however, that enzyme has evolved, *that is*, become neofunctionalized into an arginine decarboxylase (ADC), producing Agm (Fig. 2*A*) (27). Genes were expressed in *E*. *coli* BL21*speD*, deleted in *S*-adenosylmethionine decarboxylase, and therefore lacking Spd; in BL21*speG*, deleted in Spd/spermine *N*-acetyltransferase, and Spd replete, and in BL21*speB*, deleted in agmatinase, with elevated levels of Agm. To assess detection of Hspd and G44G, we analyzed benzoylated pure samples of Hspd and G44G using a SCIEX Triple Quad 6500+ LC-MS/MS system. Although the tribenzoylated Hspd (precursor ion 472.289/daughter ion 247.100) was detected as a single peak, the pentabenzoylated G44G (precursor ion 764.402/daughter ion 514.300) exhibited evidence of some isomerization, with four peaks detected. We therefore used a SCIEX TripleTOF 6600+ system, a high-resolution mass spectrometer coupled to HPLC (HR-LCMS) to analyze samples, and found single peaks for benzoylated Hspd and G44G.

**Figure 2:**
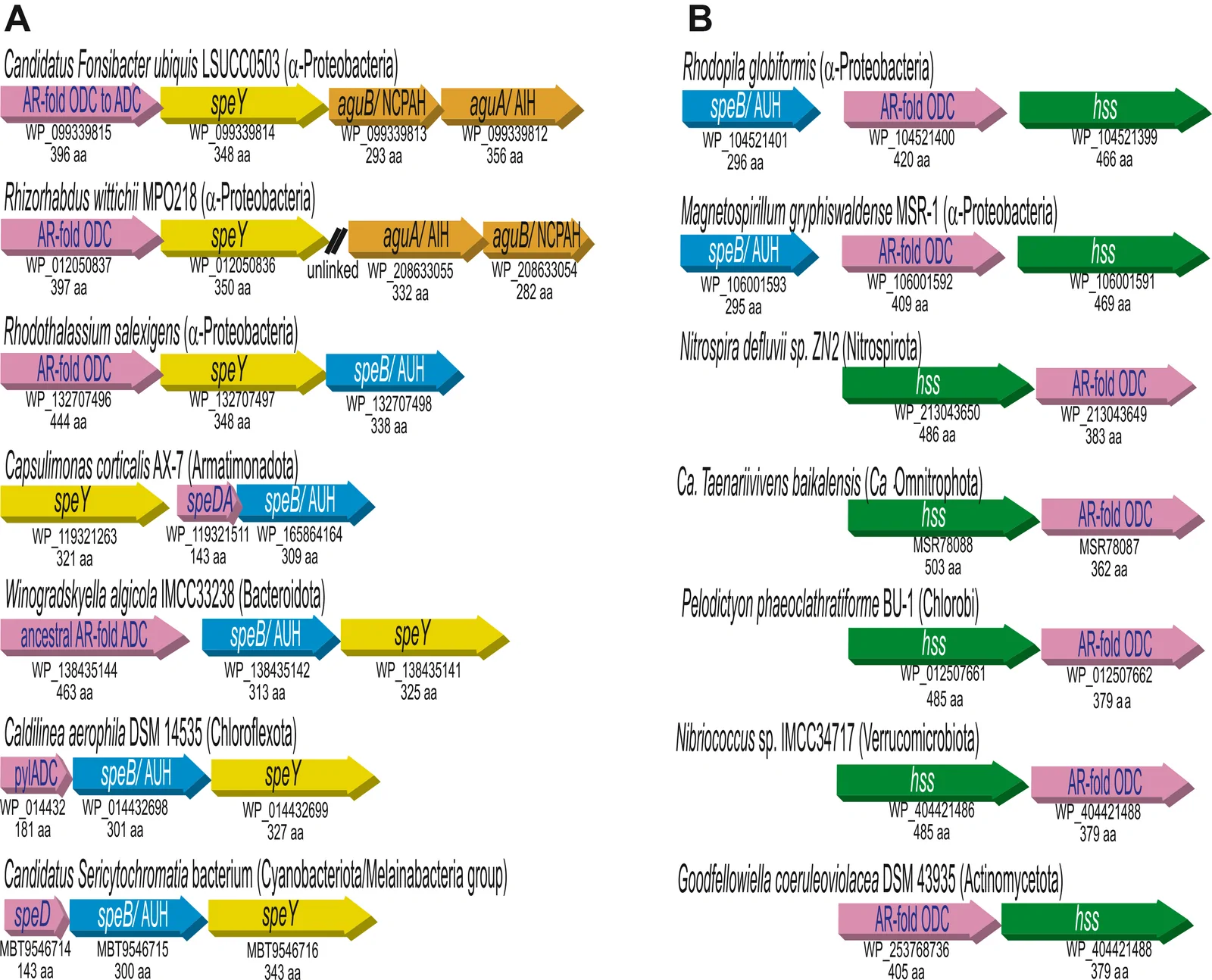
**Homospermidine biosynthesis-related gene clusters in bacteria.***A*, SpeY-encoding gene clusters. AR-fold ODC to ADC, alanine racemase-fold L-ornithine decarboxylase homolog neofunctionalized to L-arginine decarboxylase; *aguA*/AIH, agmatine deiminase/iminohydrolase; *aguB*/NCPAH, *N*-carbamoylputrescine amidohydrolase; *speB*/AUH, agmatinase/agmatine ureohydrolase; *speDA*, *S*-adenosylmethionine decarboxylase homolog neofunctionalized to L-arginine decarboxylase; ancestral AR-fold ADC, ancestral alanine racemase-fold L-arginine decarboxylase; pylADC, pyruvoyl-dependent L-arginine decarboxylase exemplified by *Thermococcus kodakarenis* pylADC; *speY*, deoxyhypusine synthase homolog. *B*, *hss*, homospermidine synthase; AR-fold ODC, alanine racemase-fold L-ornithine decarboxylase homolog.

After growth of the *E*. *coli* BL21strains in polyamine-free chemically defined M9 medium, polyamines were extracted from the pelleted cultures, and after benzoylation were analyzed by HR-LCMS. Table 1 (Group 1) shows that expression of the human and *A. thaliana dhs* genes resulted in Hspd accumulation in Spd-replete BL21*speG*, with less Hspd in BL21*speB*, and none in BL21*speD*, which lacks Spd. The differences between the calculated mass for tribenzoylated homospermidine and mass detected by the HR-LCMS, *that is*, the mass errors, are shown in Table S2. No detectable G44G was produced by the human or plant *dhs* genes in any strain. Hspd was produced by expression of the *Ca*. *F*. *ubiquis speY* gene in BL21*speD* and BL21*speG*, but none was detected in agmatinase-deficient BL21*speB*. In contrast, expressions of the *Ca*. *F*. *ubiquis speY* gene caused a large accumulation of G44G in BL21*speB*, with some detectable G44G in BL21*speG* and BL21*speD*. These data indicate that the *Ca*. *F*. *ubiquis* SpeY enzyme efficiently produces G44G from agmatine in BL21*speB* and requires the *E*. *coli* SpeB enzyme (agmatinase) to convert G44G to Hspd.

**Table 1:** High Resolution LC-MS detection of benzoylated homospermidine and G44G

DHS-, SpeY- and HSS-encoding genes in *E*. *coli*	BL21*speD* (AUP)	BL21*speG* (AUP)	BL21*speB* + arg (AUP)
Homospermidine			
Group 1			
Empty pETDuet-1	2.04 × 10^4^	1.51 × 10^4^	1.67 × 10^4^
*Arabidopsis thaliana* DHS	3.65 × 10^4^	4.91 × 10^7^	5.09 × 10^6^
*Homo sapiens* DHS	7.48 × 10^4^	3.79 × 10^7^	8.35 × 10^6^
*Ca*. *Fonsibacter ubiquis* SpeY	1.04 × 10^7^	4.46 × 10^6^	8.16 × 10^4^
Group 2			
Empty pETDuet-1	4.39 × 10^4^	4.92 × 10^4^	4.50 × 10^4^
*Caldilinea aerophila* SpeY	1.27 × 10^8^	7.78 × 10^7^	3.96 × 10^5^
*Capsulimonas corticalis* SpeY	2.38 × 10^8^	1.48 × 10^8^	8.60 × 10^5^
*Ca*. *Sericytochromatia* SpeY	1.08 × 10^8^	1.37 × 10^7^	8.18 × 10^5^
*Rhizorhabdus wittichii* SpeY	7.41 × 10^7^	1.16 × 10^7^	7.88 × 10^5^
*Winogradskyella algicola* SpeY	1.20 × 10^8^	9.48 × 10^7^	4.56 × 10^5^
Group 3			
Empty pETDuet-1	5.51 × 10^4^	4.01 × 10^4^	1.62 × 10^5^
*Magnetospirillum gryphiswaldense* HSS	8.36 × 10^8^	7.20 × 10^8^	1.53 × 10^7^
*Brucella abortus* HSS	8.32 × 10^8^	6.72 × 10^8^	3.05 × 10^7^
G44G			
Group 1			
Empty pETDuet-1	N.D.	5.28 × 10^2^	1.67 × 10^4^
*Arabidopsis thaliana* DHS	N.D.	N.D.	3.69 × 10^4^
*Homo sapiens* DHS	N.D.	N.D.	N.D.
*Ca*. *Fonsibacter ubiquis* SpeY	5.54 × 10^5^	8.39 × 10^5^	2.17 × 10^8^
Group 2			
Empty pETDuet-1	4.95 × 10^3^	3.52 × 10^4^	2.88 × 10^4^
*Caldilinea aerophila* SpeY	1.72 × 10^5^	1.30 × 10^5^	3.11 × 10^8^
*Capsulimonas corticalis* SpeY	1.52 × 10^5^	1.14 × 10^5^	3.19 × 10^8^
*Ca*. *Sericytochromatia* SpeY	6.88 × 10^5^	1.26 × 10^5^	2.60 × 10^8^
*Rhizorhabdus wittichii* SpeY	5.43 × 10^6^	8.94 × 10^5^	2.31 × 10^8^
*Winogradskyella algicola* SpeY	3.35 × 10^5^	1.94 × 10^5^	1.81 × 10^8^
Group 3			
Empty pETDuet-1	1.30 × 10^4^	3.92 × 10^3^	1.54 × 10^4^
*Magnetospirillum gryphiswaldense* HSS	6.22 × 10^3^	1.29 × 10^4^	6.70 × 10^3^
*Brucella abortus* HSS	1.32 × 10^4^	6.68 × 10^3^	1.80 × 10^4^

High Resolution Liquid Chromatography Mass Spectrometry (HR-LCMS) detection of tribenzoylated homospermidine (Hspd) (calculated mass 472.2594684) and pentabenzoylated G44G (764.355494) in cell extracts of *E*. *coli* BL21*speD*, BL21*speG* and BL21*speB* grown with 300 μM arginine (arg, Groups 1 & 2) or 2 mM arg (Group 3). *SpeY*, *dhs and hss* genes are expressed from pETDuet-1. AUP, area under peak; *Ca*., *Candidatus*. All strains within each group were grown in chemically-defined, polyamine-free M9 medium and analyzed together but each group represents an independent experiment. The *E*. *coli* BL21*speD*, *speG* and *speB* strains are single gene deletions of *S*-adenosylmethionine decarboxylase, spermidine *N*-acetyltransferase and agmatine ureohydrolase, respectively. N.D., not detected.

### Phylogenetically diverse bacterial SpeY homologs produce *N1*,*N9*-*bis*(guanidino)homospermidine

To further explore the potential diversity of G44G-based Hspd production in bacteria, we selected *speY* gene homologs found in phylogenetically diverse bacteria that also encode ADC homologs from different protein folds, *that is*, nonhomologous isofunctional enzymes (28) that convergently produce Agm from L-arginine (12, 29). The *T*. *thermus* genome encodes a typical alanine racemase-fold (AR-fold), pyridoxal 5′-phosphate (PLP) cofactor-dependent ADC, similar to the *E*. *coli* and *Vibrio vulnificus* biosynthetic ADCs (30). SpeY-encoding gene clusters containing *speY* and ADC, along with agmatinase (*speB*) or Agm deiminase/iminohydrolase (*aguA*/AIH) and *N*-carbamoylputrescine amidohydrolase (*aguB*/NCPAH), are shown in Figure 2*A*. AIH and NCPAH convert Agm to Put *via N*-carbamoylputrescine (Fig. 1*A*) (31). A homolog of the ancestral form of the AR-fold ADC (29) is found encoded along with SpeB and SpeY in a gene cluster from the Bacteroidota mesophilic species *Winogradskyella algicola* (32). Ancestral AR-fold ADC lacks the approximately 100-amino-acid four-helix bundle structure found inserted between the N- and C-terminal domains of the *E*. *coli* and *V*. *vulnificus* ADCs (29). A human-type ODC, an AR-fold PLP cofactor-dependent enzyme encoded by the α-proteobacterium mesophilic species *Rhizorhabdus wittichii* (33), is, like the *Ca*. *F*. *ubiquis* ODC, immediately upstream of *speY*, and this species encodes AIH and NCPAH in a different part of the genome. This suggests that the *R*. *wittichii* ODC has become neofunctionalized into an ADC. A gene cluster encoding SpeY, SpeB and an *S*-adenosylmethionine decarboxylase (SpeD) homolog is found in the Armatimonadota mesophilic species *Capsulimonas corticalis* (34). The *C*. *corticalis* pyruvoyl cofactor-dependent SpeD homolog was previously shown to be neofunctionalized, exhibits ADC activity and is designated SpeDA (35). Another example of a gene cluster encoding SpeY, SpeB, and a potentially neofunctionalized SpeD homolog is found in a metagenome-assembled genome of the mesophilic Cyanobacteriota/Melainabacteria nonphotosynthetic species *Ca*. *Sericytochromatia* bacterium (36). SpeY, SpeB, and a homolog of the archaeal pyruvoyl-dependent ADC (37) are found encoded in a gene cluster from the Chloroflexota filamentous thermophilic species *Caldilinea aerophila* (38). The *C*. *aerophila* pyruvoyl-dependent ADC evolved from a protein fold unrelated to the *Ca*. *Sericytochromatia* bacterium and *C*. *corticalis* pyruvoyl-dependent SpeDA ADCs.

SpeY-encoding genes in these species are found in very diverse evolutionary and ecological contexts for Agm production, and should indicate whether the use of Agm to form G44G is a common feature of bacterial Hspd biosynthesis. The various *speY* genes were expressed in *E*. *coli* BL21*speD*, BL21*speG* and BL21*speB*, and benzoylated Hspd and G44G from these strains analyzed by HR-LCMS with comparison to pure samples of Hspd and G44G (Table 1; Group 2). Hspd was produced by expression of each gene, with the accumulation of Hspd being BL21*speD*>BL21*speG*>>BL21*speB*. In contrast, *speY* genes produced a large accumulation of G44G in BL21*speB*, with considerably less in BL21*speD* and BL21*speG*. Individual chromatograms for tribenzoylated Hspd and pentabenzoylated G44G are shown in Fig. S1. The very low levels of Hspd detected in BL21*speB* indicate that the *E*. *coli* agmatinase is required to convert G44G into Hspd. Due to potential variation in the steady-state protein levels produced by each expression construct, resulting from potential variations in expression levels, exact differences in absolute amounts of Hspd and G44G between strains cannot be directly compared.

### Diverse agmatinase homologs hydrolyze *N*^*1*^,*N*^*9*^-*bis*(guanidino)homospermidine to homospermidine

To more fully investigate the role of SpeB (agmatinase) in converting G44G to Hspd, we coexpressed the bacterial *speY* genes in BL21*speB* with different *speB* genes and detected tribenzoylated Hspd using an Agilent 6130 quadrupole ESI mass spectrometer coupled to an HPLC (LC-MS) (Fig. 3). We included as a control the *speY* and *speB* genes of *T*. *thermophilus* that were previously shown to produce Hspd *via* G44G (25, 26). We also employed the *speB* gene of *Anabaena* sp. PCC 7120, a Cyanobacteriota species that requires SpeY to synthesize Hspd (24), in addition to the *speB* genes of Ca. *F*. *ubiquis*, *W*. *algicola* and *C*. *aerophila*. This coexpression experiment clearly shows that in the absence of a functional *speB* gene, essentially no Hspd is produced, and coexpression of *speY* and *speB* genes produces a large accumulation of Hspd, varying by approximately only 3.5-fold between the different *speY* and *speB* combinations (Fig. 3). An independent experiment (cultures grown and analyzed at a different time) confirmed these results (Fig. S2). It is notable that when the *T*. *thermophilus speY* was coexpressed with the *Anabaena speB*, Hspd accumulated approximately 3-fold more compared to coexpression with the *T*. *thermophilus speB*.

**Figure 3:**
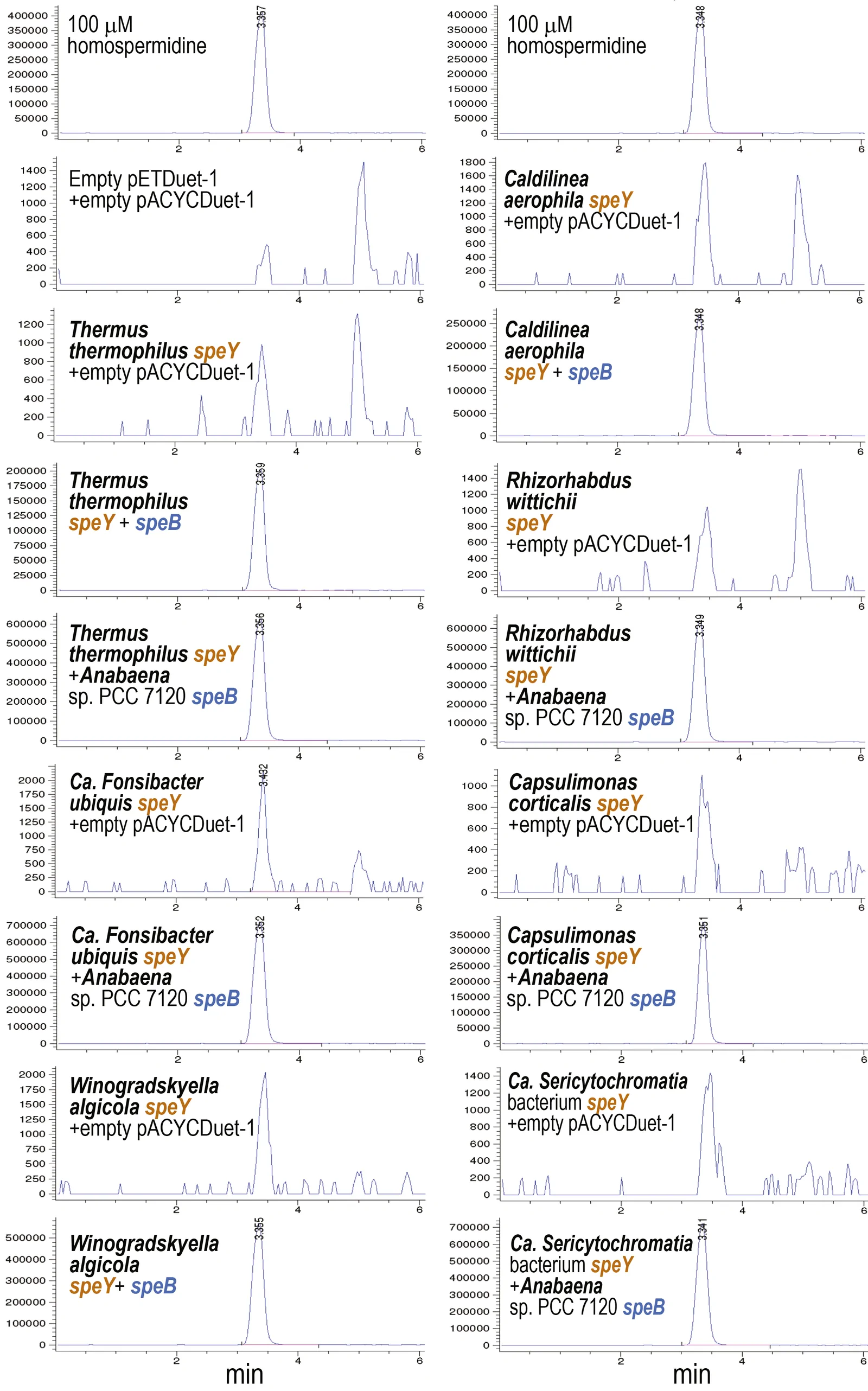
**LC-MS detection of tribenzoylated homospermidine.** SpeY-encoding genes were expressed from pETDuet-1, and SpeB homolog-encoding genes expressed from pACYCDuet-1 in *E*. *coli* BL21*speB* grown with 2.0 mM arginine. Shown are the chromatograms for the mass tolerance window 471.95:472.95. Two different samples of 100 μM pure homospermidine are shown.

Our LC-MS system was unable to detect benzoylated G44G, so to confirm that the *speY* genes did cause production of G44G, the strains were grown again in M9 medium and analyzed by HR-LCMS (Tables 2 and S3). These data confirm that Hspd is not detected above background noise when only the *speY* genes are expressed in BL21*speB*, and Hspd is produced by all *speY+speB* coexpressing strains. G44G levels in strains expressing only *speY* are similar to Hspd levels in strains coexpressing *speY* and *speB*. Individual chromatograms for Hspd and G44G from this HR-LCMS analysis are shown in Figs. S3 and S4. The *Ca*. *F*. *ubiquis*, *R*. *wittichii*, *C*. *corticalis*, *W*. *algicola and Ca*. *Sericytochromatia* bacterium *speY* genes are from mesophilic species, and we have shown that expression of these *speY* genes produces G44G in *E*. *coli*. The original trivial name given to G44G was caldomycin (25, 26). Caldomycin is a hybrid word, derived from the Latin *caldus* (warm, hot) and the Greek μυκης, meaning fungus. As *N*^1^,*N*^9^-*bis*(guanidino)-5-aza-nonane (G44G) is likely to be found in many mesophiles, “hot fungus” may not be an appropriate name for G44G. We suggest the trivial name “diagmatine”, consistent with the switch from growth temperature-to structure-based names for polyamines, for example, caldine to norspermidine, and thermine to norspermine.

**Table 2:** High Resolution LCMS detection of benzoylated G44G and homospermidine in cell extracts of *E*. *coli* BL21*speB*+ 2 mM arginine

Expressed genes	AUP 2.78 min (Hspd)	AUP 3.07 min (G44G)
pETDuet-1 + pACYCDuet-1	3.28 × 10^3^	7.77 × 10^1^
ThetheSpeY + pACYCDuet-1	4.09 × 10^3^	2.46 × 10^6^
FonubiSpeY + pACYCDuet-1	7.71 × 10^3^	2.48 × 10^6^
WinalgSpeY + pACYCDuet-1	7.15 × 10^3^	3.41 × 10^6^
CalaerSpeY + pACYCDuet-1	6.76 × 10^3^	4.34 × 10^6^
RhiwitSpeY + pACYCDuet-1	6.00 × 10^3^	3.72 × 10^6^
CapcorSpeY + pACYCDuet-1	6.38 × 10^3^	2.14 × 10^6^
CaSerSpeY + pACYCDuet-1	5.63 × 10^3^	2.98 × 10^6^
ThetheSpeY + ThetheSpeB	3.90 × 10^6^	1.02 × 10^6^
FonubiSpeY + AnabaeSpeB	7.10 × 10^6^	6.61 × 10^3^
WinalgSpeY + WinalgSpeB	6.86 × 10^6^	4.76 × 10^4^
CalaerSpeY + CalaerSpeB	4.42 × 10^6^	2.87 × 10^5^
RhiwitSpeY + AnabaeSpeB	7.82 × 10^6^	6.40 × 10^4^
CapcorSpeY + AnabaeSpeB	5.50 × 10^6^	2.71 × 10^4^
CaSerSpeY + AnabaeSpeB	7.03 × 10^6^	7.79 × 10^4^

High Resolution Liquid Chromatography Mass Spectrometry (HR-LCMS) detection of tribenzoylated homospermidine (Hspd) (calculated mass 472.2594684) and pentabenzoylated G44G (764.355494) in cell extracts of *E*. *coli* BL21*speB* grown with 2 mM arginine in M9 medium. SpeY-encoding genes are expressed from pETDuet-1 and SpeB genes from pACYCDuet-1. AUP, area under peak. All strains were grown and analyzed together. Thethe, *Thermus thermophilus*; Fonubi, *Candidatus Fonsibacter ubiquis*; Winalg, *Winogradskyella algicola*; Calaer, *Caldilinea aerophila*; Rhiwit, *Rhizorhabdus wittichii*; Capcor, *Capsulimonas corticalis*; CaSer, *Ca*. *Sericytochromatia* bacterium; Anabae, *Anabaena* sp. PCC 7120.

The *speB* homologs used in the above coexpression experiment are derived from genomes that encode SpeY: *T*. *thermophilus*, *Anabaena* sp. PCC 7120, *Ca*. *F*. *ubiquis*, *W*. *algicola* and *C*. *aerophila*. This indicates that the encoded SpeB enzymes convert G44G to Hspd. However, Table 1 shows that the *E. coli speB* gene is required for Hspd accumulation when *speY* homologs alone are expressed in BL21*speB*. The native substrate of the *E*. *coli* SpeB enzyme is Agm, which it directly converted to Put by hydrolysis of the guanidino group (39); however, the data in Table 1 show that *E*. *coli* SpeB must also be able to act on G44G. It also implies that *E*. *coli* SpeB hydrolyzes G44G to *N*^1^-guanidinohomospermidine (G44) and then hydrolyzes G44 to Hspd.

To assess the substrate flexibility of different types of SpeB proteins, we coexpressed the *C*. *corticalis speY* gene with genes encoding SpeB homologs, *that is*, ureohydrolases that likely act on different substrates. We included the SpeB-encoding genes of *T*. *thermophilus*, *Anabaena* sp. PCC 7120, *W*. *algicola*, and *C*. *aerophila*, which we have shown act on G44G, the *E*. *coli* SpeB that normally acts on Agm, and the *Bacillus subtilis* SpeB, which likely acts on *N*^1^-aminopropylagmatine (27, 40). We also included a SpeB homolog encoded by the α-proteobacterium *Magnetospirillum gryphiswaldense*, which encodes in the same gene cluster (Fig. 2*B*) a human-type ODC that is probably neofunctionalized to ADC and a bacterial homospermidine synthase (HSS). The HSS associated with the *M*. *gryphiswaldense* SpeB (Fig. 2*B*) synthesizes Hspd but not G44G (Tables 1 and S2, Group 3). This suggests that the usual substrate of the *M*. *gryphiswaldense* SpeB is agmatine. Tables 3 and S4 show that coexpression of the *C*. *corticalis speY* gene with each *speB* gene produces approximately, within a 2-fold variation, similar levels of Hspd. Individual chromatograms for Hspd from this HR-LCMS analysis are shown in Fig. S5. These data suggest that SpeB enzymes that usually act on Agm or *N*^1^-aminopropylagmatine, can also hydrolyze G44G.

**Table 3:** HR-LCMS detection of homospermidine produced by coexpression of *Capsulimonas corticalis* SpeY- and diverse SpeB-encoding ureohydrolases

Expressed genes i*n E*. *coli BL21speB*	AUP (Hspd)
pETDuet-1 + pACYCDuet-1	1.65 × 10^5^
CapcorSpeY + pACYCDuet-1	1.77 × 10^6^
CapcorSpeY + ThetheSpeB	5.57 × 10^8^
CapcorSpeY + AnabaeSpeB	7.55 × 10^8^
CapcorSpeY + WinalgSpeB	7.83 × 10^8^
CapcorSpeY + CalaerSpeB	3.82 × 10^8^
CapcorSpeY + EcoliSpeB	6.45 × 10^8^
CapcorSpeY + BacsubSpeB	4.82 × 10^8^
CapcorSpeY + MaggrySpeB	3.57 × 10^8^

HR-LCMS detection of tribenzoylated homospermidine (Hspd) (calculated mass = 472.259) in cell extracts of *E*. *coli* BL21*speB* grown without added arginine. The *speY* gene is expressed from pETDuet-1 and *speB* genes from pACYCDuet-1. AUP, area under peak; All strains were grown and analyzed together. Bacsub, *Bacillus subtilis*; Ecoli, *Escherichia coli*; Maggry, *Magnetospirillum gryphiswaldense*.

### SpeY-associated agmatinase homologs are specific *N*^*1*^,*N*^*9*^-*bis*(guanidino)homospermidine ureohydrolases

The enzymatic efficiency of the different types of SpeB enzyme with either Agm or G44G as substrates was investigated. We purified recombinant SpeB proteins produced in *E*. *coli*, including the *E*. *coli* SpeB (usual substrate Agm), *B*. *subtilis* (*N*^1^-aminopropylagmatine) and *C*. *aerophila* and *W*. *algicola* (G44G). The assay of enzymatic activity is affected by the fact that G44G presents two guanidino groups per molecule. Table 4 shows that the *E*. *coli* SpeB was the most flexible towards the Agm and G44G substrates and was approximately 2-fold more efficient (*k*_cat_/*K*_M_) with G44G compared to Agm (Table 4). Since G44G has two guanidino groups, this would suggest that *E*. *coli* barely discriminates between Agm and G44G. The *B*. *subtilis* SpeB is approximately 330-fold more efficient with G44G than with Agm, indicating that overall substrate structure is important. Notably, the SpeB proteins of *C*. *aerophila* and *W*. *algicola* exhibited no detectable activity with Agm but exhibited very similar catalytic efficiencies with G44G. The *E*. *coli* SpeB was approximately a third as active with G44G as the *C*. *aerophila* and *W*. *algicola* SpeB proteins, but surprisingly, the *B*. *subtilis* SpeB was 5-fold more efficient with G44G than were the *C*. *aerophila* and *W*. *algicola* SpeB homologs. Considering that the *C*. *aerophila* and *W*. *algicola* SpeB homologs do not act on Agm, we propose that these enzymes should be designated as diagmatine ureohydrolases (DUH) or diagmatinases.

**Table 4:** Kinetic parameters for agmatine ureohydrolase (AUH), agmatine iminohydrolase (AIH) and alanine racemase-fold ornithine decarboxylase (AR-fold ODC) homologs

Protein (concentration)	Substrate	*K*_*M*_ (mM)	*k*_cat_ (s^−1^)	*k*_cat_/*K*_*M*_ (s^−1^ M^−1^)
AUH				
*E*. *coli* (200 nM)	agm	3.71 ± 0.19	18.54 ± 0.22	5.0 × 10^3^ ± 2.0 × 10^2^
*E*. *coli* (200 nM)	G44G	0.57 ± 0.20	7.13 ± 0.61	1.3 × 10^4^ ± 2.9 × 10^3^
*Bacillus subtilis* (200 nM)	agm	3.78 ± 0.19	2.22 ± 0.02	5.9 × 10^2^ ± 2.3 × 10^1^
*Bacillus subtilis* (200 nM)	G44G	0.46 ± 0.01	91.83 ± 0.49	2.0 × 10^5^ ± 2.7 × 10^3^
*Caldilinea aerophila* (200 nM)	agm	N.D.	N.D.	N.D.
*Caldilinea aerophila* (100 nM)	G44G	0.77 ± 0.04	31.90 ± 0.20	4.1 × 10^4^ ± 2.0 × 10^3^
*Winogradskyella algicola* (200 nM)	agm	N.D.	N.D.	N.D.
*Winogradskyella algicola* (100 nM)	G44G	0.58 ± 0.004	24.28 ± 0.15	4.2 × 10^4^ ± 5.0 × 10^2^
AIH				
*Pseudomonas aeruginosa* (50 nM)	agm	1.04 ± 0.03	17.28 ± 0.20	1.7 × 10^4^ ± 2.7 × 10^2^
*Pseudomonas aeruginosa* (50 nM)	G44G	0.14 ± 0.04	0.77 ± 0.06	5.7 × 10^3^ ± 1.1 × 10^3^
*Rhizorhabdus wittichii* (100 nM)	agm	N.D.	N.D.	N.D.
*Rhizorhabdus wittichii* (100 nM)	G44G	0.10 ± 0.001	7.81 ± 0.11	7.7 × 10^4^ ± 2.0 × 10^3^
AR-fold ODC				
*Brucella abortus* (100 nM)	orn	0.76 ± 0.01	3.11 ± 0.03	4.1 × 10^3^ ± 3.5 × 10^0^
*Brucella abortus* (100 nM)	arg	N.D.	N.D.	N.D.
*Rhodothalassium salexigens* (100 nM)	orn	N.D.	N.D.	N.D.
*Rhodothalassium salexigens* (100 nM)	arg	1.69 ± 0.04	5.62 ± 0.10	3.3 × 10^3^ ± 1.2 × 10^2^
*Rhodopila globiformis* (50 nM)	orn	N.D.	N.D.	N.D.
*Rhodopila globiformis* (50 nM)	arg	2.20 ± 0.10	4.99 ± 0.05	2.3 × 10^3^ ± 1.3 × 10^2^

Purified N-his-tagged proteins assayed at pH 7.5 and 25 °C for AUH, 30 °C for AIH, and 25 °C for AR-fold ODC (±S.D. of three replicate assays). *E*. *coli* AUH [GenBank: BAE77000; 306 aa], *B*. *subtilis* AUH [CAB15776; 290 aa], *C*. *aerophila* AUH [WP_014432698; 301 aa], *W*. *algicola* AUH [WP_138435142; 313 aa], *P*. *aeruginosa* AIH/AguA [NP_248983; 368 aa], *R*. *wittichii* AIH [WP_208633055; 332 aa], *B*. *abortus* AR-fold ODC [WP_006121644; 377 aa], *R*. *salexigens* AR-fold ODC/ADC [WP_132707496; 444 aa], *R*. *globiformis* AR-fold ODC/ADC [WP_104521400; 420 aa]. agm, agmatine; arg, L-arginine; G44G, caldimycin/diagmatine; N.D. (no detectable activity); orn, L-ornithine.

### Diverse agmatine deiminase/iminohydrolase homologs hydrolyze *N*^*1*^,*N*^*9*^-*bis*(guanidino)homospermidine to *N*^*1*^,*N*^*9*^-*bis*(carbamoyl)homospermidine

An alternative to agmatinase/SpeB for producing Put from Agm employs Agm deiminase/iminohydrolase (AIH/AguA) to convert Agm to *N*-carbamoylputrescine, and *N*-carbamoylputrescine amidohydrolase (NCPAH/AguB) to convert *N*-carbamoylputrescine to Put, as found in *Pseudomonas aeruginosa* (31, 41). A different pathway uses AIH to produce *N*-carbamoylputrescine, then Put transcarbamoylase converts *N*-carbamoylputrescine to Put and carbamoylphosphate. This degradative pathway is known as the Agm deiminase pathway, associated with an Agm-Put antiporter activity, and is found in species such as *Enterococcus faecalis* (Bacillota phylum) (42, 43). The AIH homologs of *Desulfosporinus orientus*, a sulfate-reducing anaerobe (Bacillota) (44) and *Rhodothermus marinus*, a thermophilic aerobe (Rhodothermota) (45), are likely to be *N*^1^-aminopropylagmatine iminohydrolases (27). Similarly, the AIHs of *Hydrogenimonas thermophila*, a thermophilic anaerobe (Campylobacterota), and the human gut commensals *Akkermansia muciniphila* (Verrucomicrobiota) (46) and *Bacteroides thetaiotaomicron* (Bacillota) (47) are likely to act on *N*^1^-aminopropylagmatine, albeit produced *via* carboxyaminopropylagmatine, similarly to *Clostridium leptum* (48). Some bacteria that encode SpeY also encode AIH and NCPAH homologs (Fig. 2*A*).

Agm deimination converts the guanidino group to an *N*-carbamoyl moiety, and for G44G, deimination of both guanidino groups is predicted to produce the previously unknown *N*^1^,*N*^9^-*bis*(carbamoyl)-5-aza-nonane, *that is*, *N*^1^,*N*^9^-*bi*s(carbamoyl)homospermidine (C44C). We used HR-LCMS to detect an exact mass for underivatized C44C (detected mass range 246.1924–246.1930, calculated mass 246.192) when *speY* and AIH-encoding homologs from either *Ca*. *F*. *ubiquis* or *R*. *wittichii* were coexpressed in *E*. *coli* BL21*speB*. When *speY* was expressed alone, no C44C was detected (Table 5, Group 1), individual chromatograms are shown in Fig. S6. In contrast, when *speY* and AIH-encoding homologs were coexpressed, an exact mass for C44C was clearly detected. We wondered whether other AIH-encoding genes from species that do not produce G44G and Hspd could produce C44C when coexpressed with the *C*. *corticalis speY* gene in BL21*speB* (Table 5, Group 2). Coexpression of each AIH/AguA-encoding gene from *P*. *aeruginosa*, *E*. *faecalis*, *Desulfosporosinus orientis*, *R*. *marinus*, *A*. *muciniphila*, *H*. *thermophila* and *B. thetaiotaomicron* resulted in the accumulation of C44C with all AIH homologs. Although the different genes cannot be compared quantitatively due to potential differences in expression levels, there is an indication that AIH proteins that are likely to act on *N*^1^-aminopropylagmatine or G44G might be more active in producing C44C than AIH proteins that normally act on Agm (Table 5).

**Table 5:** HR-LCMS detection of unbenzoylated *N*^1^,*N*^9^-*bis*(*N*-carbamoyl) homospermidine in *E*. *coli* cell extracts

Expressed in *E*. *coli* BL21*speB*	AUP	Detected mass
Group 1 (with 2 mM arginine)		
100 μM caldimycin	ND	ND
pETDuet-1 + pACYCDuet-1	ND	ND
FunubiSpeY + pACYCDuet-1	ND	ND
FunubiSpeY + FunubiAIH	1.19 × 10^8^	246.1926
RhiwitSpeY + pACYCDuet-1	ND	ND
RhiwitSpeY + RhiwitAIH	8.31 × 10^7^	246.1924
Group 2 (no added arginine)		
pETDuet-1 + pACYCDuet-1	ND	ND
CapcorSpeY + pACYCDuet-1	ND	ND
CapcorSpeY + PseaerAIH	3.44 × 10^6^	246.1924
CapcorSpeY + EntfaeAIH	1.10 × 10^7^	246.1926
CapcorSpeY + DesoriAIH	1.02 × 10^8^	246.1925
CapcorSpeY + RhomarAIH	8.67 × 10^7^	246.1931
CapcorSpeY + AkkmucAIH	8.77 × 10^7^	246.1932
CapcorSpeY + HydtheAIH	6.81 × 10^7^	246.1933
CapcorSpeY + BactheAIH	9.47 × 10^7^	246.1930

High Resolution LCMS (HR-LCMS) detection of underivatized *N*^1^,*N*^9^-*bis*(*N*-carbamoyl)homospermidine (calculated mass 246.192) in cell extracts of *E*. *coli* BL21*speB* grown with or without added arginine. SpeY genes are expressed from pETDuet-1 and AIH-encoding genes from pACYCDuet-1. AIH, agmatine deiminase/iminohydrolase. AUP, area under peak; All strains within Group 1 and Group 2 were grown and analyzed together, but Group 1 and 2 were independent experiments. Akkmuc, *Akkermansia muciniphila*; Bacthe, *Bacteroides thetaiotaomicron*; Desori, *Desulfosporosinus orientis*; Entfae, *Enterococcus faecalis*; Hydthe, *Hydrogenimonas thermophila*; Pseaer, *Pseudomonas aeruginosa*; Rhomar, *Rhodothermus marinus*.

### A SpeY-associated agmatine iminohydrolase homolog is a specific *N*^*1*^,*N*^*9*^-*bis*(guanidino)homospermidine iminohydrolase

To investigate this potential difference in substrate preference, we purified the *P*. *aeruginosa* recombinant canonical AIH expressed in *E*. *coli*. This is a biochemically validated Agm deiminase producing *N*-carbamoylputrescine. We also purified the AIH homolog from *R*. *wittichii* expressed in *E*. *coli*, which we predict is likely to act on G44G. Enzymatic activity of each protein was assayed with either Agm or G44G as substrate (Table 4). With the *P*. *aeruginosa* AIH, the binding affinity (*K*_M_) for G44G was 7-fold greater than for Agm, however, the turnover (*k*_cat_) of Agm was 22 times faster than with G44G. Consequently, the enzymatic efficiency of the *P*. *aeruginosa* AIH protein was 3-fold greater with Agm than with G44G. In contrast, the *R*. *wittichii* AIH homolog was not detectably active with Agm, and its enzymatic efficiency with G44G was 13.5-fold higher than that of the *P*. *aeruginosa* AIH with G44G (Table 4). The *R*. *wittichii* AIH/AguA homolog is therefore specifically a G44G (diagmatine) deiminase/iminohydrolase, *that is*, *N*^1^,*N*^9^-*bis*(guanidino)-5-aza-nonane deiminase/iminohydrolase, for which we propose the trivial name of diagmatine iminohydrolase (DIH).

### Neofunctionalized ornithine decarboxylases in homospermidine biosynthesis

Biosynthesis of Hspd by the SpeY pathway appears to be universally dependent on Agm and G44G in bacteria and therefore requires the presence of ADC to produce Agm from L-arginine. In contrast, biosynthesis of Hspd by the HSS enzyme in bacteria universally depends on Put, which can be formed from the ODC or ADC routes. We noticed that in diverse bacteria, the *hss* gene is adjacent to an AR-fold ODC-encoding gene, indicating that Hspd biosynthesis involves L-ornithine decarboxylation to form Put, which is then converted to Hspd by HSS (Fig. 2*B*). However, in some bacteria, an agmatinase-encoding *speB* gene is immediately upstream of the ODC-encoding gene, suggesting that the ODC gene may have become neofunctionalized to an ADC (Fig. 2*B*). We selected the AR-fold ODC of the HSS-encoding α-proteobacterium *Rhodophila globiformis*, from the Acetobacterales order, to test for ODC and ADC activity. As a comparison, we analyzed the enzymatic activity of the α-proteobacterium *Brucella abortus* AR-fold ODC (Hyphomicrobiales order). This species encodes an HSS that produces Hspd but not G44G (Table 1, Group 3), indicating that agmatine is not part of the Hspd biosynthetic pathway. Purified recombinant *R*. *globiformis* and *B*. *abortus* proteins were assayed with L-arginine and L-ornithine (Table 4). The *B*. *abortus* ODC homolog exhibited enzymatic activity only in the presence of L-ornithine, and the *R*. *globiformis* ODC homolog exhibited activity only in the presence of L-arginine. The catalytic efficiency (*k*_cat_/*K*_M_) of the *B*. *abortus* protein with L-ornithine was approximately 2-fold higher than the *R*. *globiformis* protein with L-arginine (Table 4). These two proteins exhibit 33% amino acid identity.

Previously, we demonstrated (49) that the SpeY-encoding α-proteobacterium species *Ca*. *Fonsibacter ubquis* possesses an AR-fold ODC homolog that is active only on L-arginine. There are multiple examples in the α-Proteobacteria of SpeY-encoding species that possess an AR-fold ODC, suggesting that these ODC homologs have also been neofunctionalized to ADC. An interesting example is the α-proteobacterium *Rhodothalassium salexigens* (Rhodothalassiales order), which is very unusual in that it accumulates *N*^1^-aminopropylhomospermidine as its most abundant polyamine (50). The *R*. *salexigens speY* gene is in a cluster with an AR-fold ODC-encoding gene and a *speB* homolog (Fig. 2*A*). We purified the recombinant ODC homolog of *R*. *salexigens* and assayed the protein with L-ornithine and L-arginine. As expected, the *R*. *salexigens* ODC homolog exhibited no detectable activity with L-ornithine but exhibited a similar catalytic efficiency with L-arginine as the *R*. *globiformis* ODC homolog (Table 4), to which it exhibits 49% amino acid identity. Thus, AR-fold ODC homologs have evolved into ADCs in species encoding SpeY, as well as in species encoding HSS and agmatinase. Our TBLASTN analysis of eubacterial genomes with a fusion protein of SpeY and AR-fold ODC indicates that these two proteins co-occur only in α-Proteobacteria and some γ-Proteobacteria species of *Coxiella*, including *C*. *burnetii*, the causative agent of Q fever.

## Discussion

At the outset of our study, it was known that SpeY was essential for Hspd biosynthesis in the cyanobacterium *Anabaena* sp. PCC 7120 (24), however, it was not known how SpeY functioned mechanistically. Recently, the SpeY homolog in hyperthermophile *T*. *thermophilus* was shown to couple two molecules of Agm to form G44G. It remained to be proved whether this mechanism found in a hyperthermophile would be the same for all bacterial SpeY homologs. We found that neither the eukaryotic DHS nor the bacterial HSS proteins synthesize G44G. In contrast, we found that all tested bacterial SpeY proteins synthesize and accumulate G44G in *E*. *coli* that lacks agmatinase/SpeB. SpeY-encoding genes expressed in *E*. *coli* produce Hspd only in the presence of the *E*. *coli* SpeB. We can conclude that diverse bacterial SpeY proteins, originating from diverse phyla and genomes that encode different, nonhomologous forms of ADC, all couple two molecules of Agm to form G44G, and that the *E*. *coli* agmatinase can convert G44G to Hspd. As the Agm-dependent bacterial SpeY and Spd-dependent eukaryotic DHS were likely acquired from archaea, the question arises as to whether archaea encode both Agm- and Spd-dependent DHS proteins.

In *E*. *coli* BL21*speB* strains that lack agmatinase but coexpress *speY* and different *speB* genes, G44G is hydrolyzed to Hspd. In fact, any SpeB homolog appears able to hydrolyze G44G, including the SpeB protein of *B*. *subtilis*, which likely normally acts on *N*^1^-aminopropylagmatine, and the SpeB of *E*. *coli*, which normally acts on Agm. Kinetic analysis of the purified recombinant SpeB proteins from *E*. *coli*, *B*. *subtilis*, *C*. *aerophila* and *W*. *algicola*, the latter two of which likely act normally on G44G, showed that whereas the *E*. *coli* SpeB is similarly active with Agm and G44G, the *B*. *subtilis* protein is over 300-fold more active with G44G than Agm. In contrast, the *C*. *aerophila* and *W*. *algicola* SpeB proteins are active with G44G but exhibit no detectable activity with Agm, and are therefore specifically *N*^1^,*N*^9^-*bis*(guanidino)homospermidine ureohydrolases, for which we proposed the trivial name DUH (Fig. 4). Similarly, AIH homologs from species where AIH would act on agmatine, *N*^1^-aminopropylagmatine or G44G, when coexpressed with SpeY in BL21speB, were all able to produce *N*^1^,*N*^9^-*bis*(carbamoyl)homospermidine (C44C) from G44G. A kinetic analysis of the purified recombinant AIH protein from *P*. *aeruginosa*, where AIH would normally convert Agm to *N*-carbamoylputrescine, and from *R*. *wittichii*, whose AIH is likely to normally convert G44G to C44C, showed that the *R*. *wittichii* AIH is specifically a G44G deiminase/iminohydrolase. The *P*. *aeruginosa* AIH/AguA is able to act on both Agm and G44G, although more efficiently with Agm. We proposed the trivial name of diagmatine iminohydrolase (DIH) for the *R*. *wittichii* AIH-like homolog (Fig. 4). Due to a lack of pure C44C, we were unable to determine if the NCPAH/AguB proteins encoded by species that also encode SpeY, are specific for C44C or are also able to hydrolyze *N*-carbamoylputrescine.

**Figure 4:**
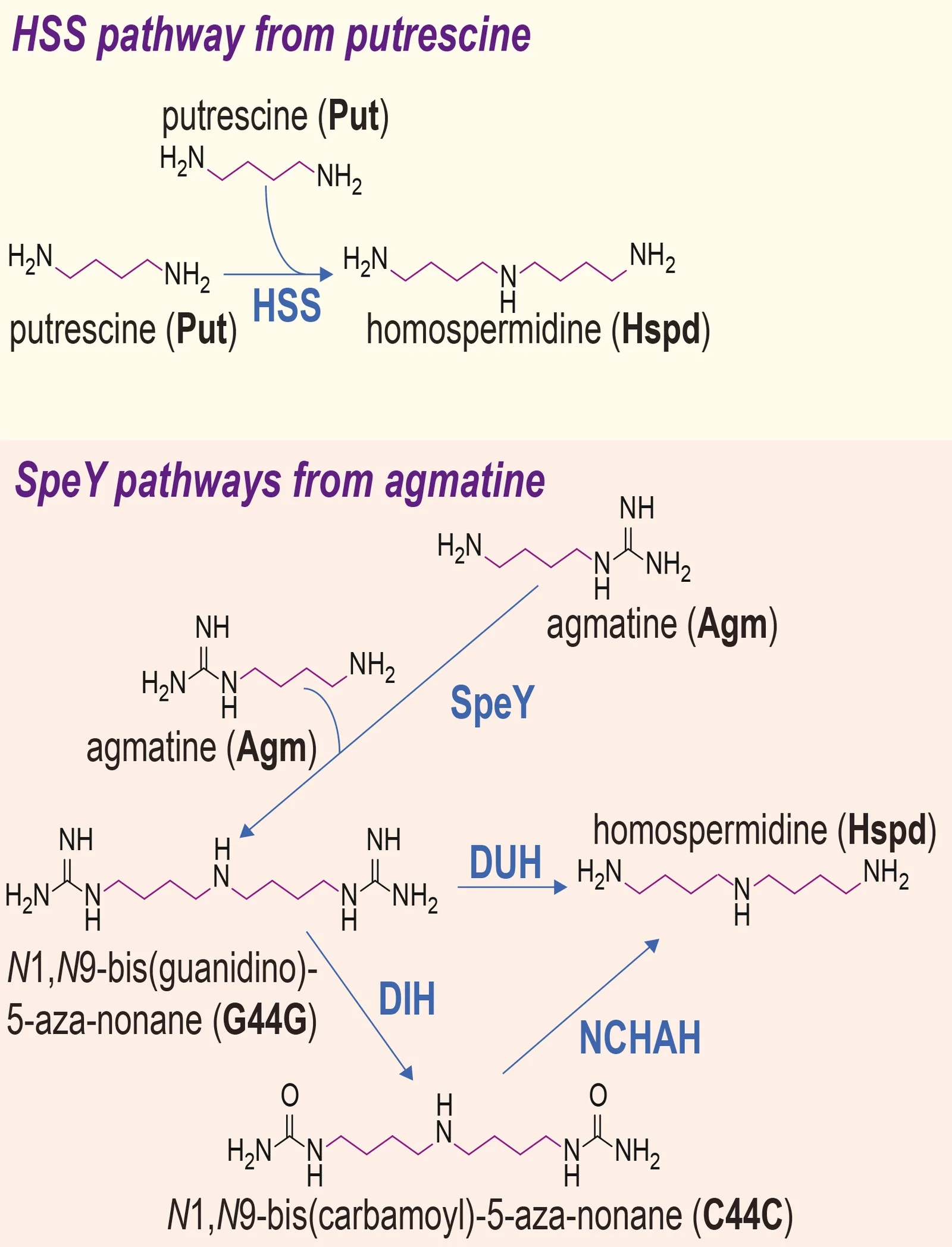
**Putrescine- and agmatine-dependent pathways for homospermidine biosynthesis.** HSS, homospermidine synthase; SpeY, *N*^1^,*N*^9^-*bis*(guanidino)5-aza-nonane synthase (diagmatine synthase); DUH, diagmatine ureohydrolase (diagmatinase); DIH, *N*^1^,*N*^9^-*bis*(guanidino)5-aza-nonane iminohydrolase (diagmatine iminohydrolase/deiminase); NCHAH, *N*^1^,*N*^9^-*bis*(carbamoyl)5-aza-nonane (*N*^1^,*N*^9^-*bis*(carbamoyl)homospermidine/C44C) amidohydrolase.

Bacterial Hspd biosynthesis appears to be divided into exclusively Put- (HSS) or Agm- (SpeY) dependent pathways (Fig. 4). However, some species encode enzymes that appear to contradict the Put- or Agm-dependence. For example, a number of SpeY-encoding α-Proteobacteria encode an AR-fold ODC, and no obvious ADC to produce Agm. We found that the AR-fold ODC of *R. thalassium*, a SpeY-encoding species, is active with L-arginine but not with L-ornithine, as was found for the AR-fold ODC of *Ca*. *F*. *ubiquis* (49). It also encodes an AUH/DUH homolog in the same gene cluster as the AR-fold ODC and SpeY. We can tentatively conclude that in bacteria encoding SpeY, an encoded AR-fold ODC homolog is likely to be neofunctionalized to ADC, and this co-occurrence is limited to α-Proteobacteria and *Coxiella* species in the γ-Proteobacteria. Similarly, the HSS-encoding species *R*. *globiformis*, besides encoding an AR-fold ODC that would be expected to produce Put directly from ornithine, paradoxically also encodes an agmatinase homolog in the same gene cluster as the *hss* and *odc* genes, suggesting the presence of ADC activity. Kinetic analysis confirmed that the *R*. *globiformis* AR-fold ODC has no activity with L-ornithine and is specifically an ADC. It seems counterintuitive that a pathway that requires Put for Hspd biosynthesis uses an ODC homolog, which would be expected to produce Put directly from L-ornithine, but in fact possesses only ADC activity. This then requires the activity of an agmatinase to convert Agm to Put. A similar arrangement of *odc*, *speB*, and *hss* is found in the α-proteobacterial species *M*. *gryphiswaldense*, and expression of its *hss* gene in *E*. *coli* does not produce G44G. Instead, it produces Hspd in the absence of the *E*. *coli* agmatinase, confirming its Put-dependence. ODC homologs neofunctionalized to ADCs may indicate that in some species, for whatever reason, ornithine is limiting.

The phylogenetically diverse but patchy distribution of *speY* in bacteria indicates complex patterns of HGT. Strict substrate specificity of DUH and DIH for G44G suggests that the transferred module during HGT is either *speY* + *duh* or *speY* + *dih* (and C44C amidohydrolase). If *speY* was transferred alone into a genome encoding ADC and canonical agmatinase/AUH, the Agm in the corresponding cell would be hydrolyzed by the AUH to Put, limiting the ability of the acquired SpeY to produce G44G and Hspd. That is, the SpeY pathway cannot function efficiently with a canonical agmatinase because the SpeY substrate would be removed by the agmatinase. The lack of activity of DUH and DIH with Agm suggests that in genomes encoding ADC, SpeY, and DUH or DIH, very little Put should be accumulated unless a separate ODC is encoded. SpeY must have agmatine as a substrate, whereas HSS must have putrescine (or two molecules of Spd). Hence, the SpeY and HSS pathways are mutually exclusive. The HSS/putrescine-dependent pathway for Hspd biosynthesis in bacteria may have evolved to accommodate the absence of ADC/Agm in some species, with HSS allowing Hspd biosynthesis without Agm. SpeY is phylogenetically more widespread than HSS, probably reflecting the fact that the ADC pathway is far more prominent than the ODC pathway among bacterial phyla. Division of Hspd biosynthesis into Put- or Agm-dependent pathways indicates that the previously understood configuration of polyamine biosynthetic pathways in bacteria requires restructuring. It is now clear that in bacteria Put is less prominent than previously understood and is absent in diverse polyamine biosynthetic pathways. Besides the diagmatine pathway, Put is also excluded from decarboxylated *S*-adenosylmethionine-dependent Spd biosynthesis from Agm *via N*^1^-aminopropylagmatine (27, 40, 51) and aspartate β-semialdehyde-dependent Spd biosynthesis from Agm *via* carboxyaminopropylagmatine (48, 52), and even from the biosynthesis of *N*-citrylputrescine in some bacterial species (53).

## Experimental procedures

### Chemicals and reagents

Agm (cat. No. A7127-5G) was purchased from Sigma-Aldrich. Hspd was a kind gift of Patrick Woster. *N* (1),*N* (9)-*bis*(guanidino)-5-aza-nonane (G44G) was custom synthesized by Wuxi AppTec. L-arginine and L-ornithine were obtained from Sigma-Aldrich. The pETDuet-1 and pACYCDuet-1 expression plasmids were purchased from Novagen. Synthetic genes with *E*. *coli*-optimized codons were purchased from GenScript. All proteins analyzed in this study are described in Table S1.

### Bacterial strains, growth and gene expression

The *E*. *coli* BL21 (DE3) strains BL21*speD* (gene deletion of *S*-adenosylmethionine decarboxylase), BL21*speG* (gene deletion of spermidine/spermine *N*-acetyltransferase) and BL21*speB* (gene deletion of agmatinase) were described previously (48). Conditions for growth in M9 chemically-defined, polyamine-free medium and gene expression induction were described previously (27). Genes were inserted into the 5′-Nde1, 3′-Xho1 sites of pETDuet-1 and pACYCDuet-1.

### Protein expression and purification

Agmatinase homolog- and agmatine deiminase homolog-encoding genes in pET28a were transformed into *E*. *coli* BL21 (DE3), and a single colony was inoculated into 40 ml of LB medium with 50 μg/ml kanamycin and grown overnight at 37 °C. Twenty-five ml of the overnight culture was then inoculated into 2 L of LB medium with 50 μg/ml kanamycin and grown to an OD_600_ of approximately 0.4. At this point, 0.2 mM isopropyl-β-d-thiogalactopyranoside (IPTG) was added to induce expression overnight at 16 °C, and the culture was then centrifuged to harvest cells. Pelleted cells were then resuspended in 20 mM Tris-HCl pH 7.5, 100 mM NaCl, 10 mM MgCl_2_, 1 mM EDTA, 0.5% NP-40 cell lysis buffer (ThermoFisher) and protease cocktail inhibitors I and II (Sigma). Resuspended cells were lysed in a cell disruptor at 10,000 p.s.i., and the lysate was centrifuged for 60 min to remove unbroken cells, debris and insoluble material. The soluble sample was applied to a 5 ml His-Trap FF (Cytiva Life Science) column equilibrated with 0.1 M NiSO_4_ and buffer A (20 mM Tris-HCl pH 7.5, 300 mM NaCl, 10 mM imidazole), then washed with 2% buffer B (20 mM Tris-HCl pH 7.5, 300 mM NaCl and 500 mM imidazole) over 10 column volumes. Proteins were then eluted from the column with a gradient of 2 to 60% buffer B over 20 column volumes and desalted by dialysis against dialysis buffer (20 mM Tris-HCl pH 7.5, 300 mM NaCl) at 4 °C overnight. Protein purity was assessed using dodecyl sulfate-polyacrylamide gel electrophoresis. Protein concentration was determined with a Biotek Synergy Multi-Mode Microplate reader at OD_280_, with the molecular weight and protein extinction coefficients program.

Preparation of the ADC/ODC decarboxylase proteins was similar to the AUH/AIH proteins. However, after growth, the pelleted cells were resuspended in a different buffer A (100 mM HEPES pH 8.0, 50 mM NaCl, 5 mM imidazole, 20 μM pyridoxal 5′-phosphate, 0.02% Brij35) containing protease inhibitor cocktails PICI and PICII. The disrupted cells were applied to a 5 ml His-Trap FF column equilibrated with 0.1 M NiSO_4_ and buffer A, then washed with 2% buffer B (100 mM HEPES buffer, pH 8.0, 50 mM NaCl, 1.0 M imidazole, 20 μM PLP, 0.02% Brij35), over 20 column volumes. Proteins were eluted from the column with a gradient of 2 to 50% buffer B over 20 column volumes. Proteins were desalted by dialysis against dialysis buffer (100 mM HEPES buffer, pH 8.0, 50 mM NaCl, 20 μM PLP, 0.02% Brij35) at 4 °C overnight.

### Agmatinase activity *in vitro* assays

The AUH/SpeB activity assay employed colorimetric detection of urea using EnzyChrom Urea Assay Kit III from Bioassay Systems. A range of enzyme concentrations between 20 nM to 2.0 μM was mixed into a buffer containing 20 mM Tris-HCl pH 7.5, 100 μM MnCl_2_, 200 mM NaCl, 1 0.0 mM dithiotheitol, and 0 to 10 mM G44G or 0 to 15 mM Agm in a total volume of 200 μl. Twenty μl of each reaction were mixed with 180 μl of urea assay buffer, 1.0 μl enzyme provided by the kit, 1 μl ketoglutarate, 8 μl reconstituted NADH reagent, 1 μl urease. One hundred μl of this mix was placed in each well in a 96-well plate, then the plate was placed in the plate reader and read at OD_340_. The temperature was set at 25 °C, 20 s per read, for 40 min. Each sample was assayed at least in triplicate. Urea production was measured by detecting absorbance at OD_340._ The concentration of urea produced was calculated by comparison to a urea standard curve from 0 to 100 μM urea using the same buffer and reagent. Sample background value was calculated in the absence of G44G or Agm. Kinetic values were calculated using the plate reader software Gen5, and *K*_m_ and *k*_cat_ were calculated using GraphPad Prism 10, employing the Michaelis-Menten model graph fitting, inputting substrate concentration and reaction velocity to calculate *K*_m_ and V_max_, and including the enzyme concentration to produce *k*_cat_ values.

### Agmatine deiminase/iminohydrolase *in vitro* assays

Activity determination is based on detection of the release of NH_3_ from Agm or G44G. The reaction contained 50 mM MES-NaOH pH 7.0 buffer, 1 mM dithiothreitol, 0 to 10 mM Agm or G44G, and 0 to 500 nM AIH enzyme at 30 °C for *R*. *wittichii* AIH, or room temperature for *P*. *aeruginosa* AIH. One hundred μl of the reaction was added to 100 μl indophenol blue reagent (0. 33 M sodium phenolate, prepared fresh before use), and 0.01% (w/v) sodium nitroprusside (prepared freshly before use), and to this mix 1 μl 10% sodium hypochlorite was added, followed by immediate mixing. After the reaction, the solution was heated to 100 °C for 5 min, and then placed on ice for 5 min. The reactions were then transferred to a 96-well plate at 200 μl per well and read at OD_640_ with a BioTek Cytation 5 plate reader. A standard curve was generated using 0 to 40 μg/ml NH_4_Cl employing the same procedure. The substrates produce a background signal at OD_640_; therefore, the substrate background was removed from the reaction absorbance data at the corresponding substrate concentration, and pure NH_4_Cl was used to generate the standard curve of OD_640_ absorbance.

### Decarboxylase *in vitro* assay

Decarboxylase assays were performed in a 200 μl solution containing 50 mM HEPES pH7.7, 100 mM NaCl, 1 mM dithiotheitol, to which was added 150 μl of carbon dioxide assay solution (Diazyme), 0 to 10 mM L-arginine or L-ornithine, and between 0 to 500 nM enzyme. The assay output was measured with a Biotek plate reader at OD_340._ The reaction rate in Absorbance Units (AU)/min was transformed to mM NADH/min using an extinction coefficient of 6.349 AU/mM/cm NADH (54) and an approximate path length of 0.58 cm for assays performed in 200 μl final volume in a Corning Costar flat bottom 96-well plate.

### Polyamine extraction and benzoylation

Cell growth, polyamine extraction and benzoylation have been previously described (55).

### Liquid chromatography-mass spectrometry (LC-MS)

An Agilent 1290 Infinity HPLC system fitted with an Eclipse XDB-C18 column (4.6 × 150 mm, 5 μm particle size), coupled to an Agilent 6130 quadrupole ESI mass spectrometer run in positive mode, was used to analyze benzoylated samples and employed a scan range of 100 to 1100 m/z. A flow rate of 0.5 ml/min at 20 °C was used for the liquid chromatography stage, with a 5 μl injection volume, employing a gradient elution with aqueous acetonitrile containing 0.1% formic acid. The benzoylation of G44G did not produce a detectable peak in the LC-MS analysis.

### Liquid chromatography-tandem mass spectrometry (LC-MS/MS)

Targeted LC-MS/MS analysis of benzoylated samples was described previously (55). In brief, reverse-phase chromatography was performed using an ACE 3 C18-PFP 150 × 4.6 mm, 3 μm HPLC column (Mac-Mod). This was coupled to an AB Sciex QTRAP 6500+ mass spectrometer equipped with an ESI ion spray source, used in positive ion mode. Data processing was achieved with SCIEX MultiQuant 3.0.3 software (AB Sciex) with relative quantification based on the peak area of each metabolite. In the current analysis, due to some detectable retention of pentabenzoylated G44G on the HPLC column, any peak found in the blank control samples was subtracted from the peak area of the preceding sample.

### High-resolution liquid chromatography-mass spectrometry (HR-LCMS)

The conditions for HR-LCMS were described previously (48). Untargeted mass spectrometric analyses were performed on a Sciex TripleTOF 6600 system (AB SCIEX) equipped with an electrospray ionization source used in the positive ionization mode.

### Gene identification

The *Anabaena* sp. PCC 7120 SpeY, *A*. *thaliana* and *Homo sapiens* DHS amino acid sequences were used to identify bacterial homologs using BLASTP (Table S1). Similarly, bacterial HSS homologs were identified using the *Agrobacterium tumefaciens* HSS amino acid sequence with BLASTP.

## Data availability

All data are contained within the manuscript and Supporting Information.

## Supporting information

This article contains supporting information.

## Conflict of interest

The authors declare that they have no conflicts of interest with the contents of this article.
