# Supplementary material for: Mutually exclusive pathways for biosynthesis of polyamine homospermidine dependent on putrescine or agmatine
Source: J Biol Chem. 2026 Sep 23;302(10):113510. doi: 10.1016/j.jbc.2026.113510 (PMC13625781; doi:10.1016/j.jbc.2026.113510)
Supplement: Supplementary file 1 [file mmc1.pdf]

**Supporting Information**

# Mutually exclusive pathways for biosynthesis of polyamine homospermidine dependent on putrescine or agmatine

Bin Li[1], Hamid R. Baniasadi[1], Jue Liang[1], Margaret A. Phillips[1] and Anthony J. Michael[1]*

[1]Department of Biochemistry, UT Southwestern Medical Center, Dallas, Texas, USA

*For correspondence: Anthony J. Michael, anthony.michael@utsouthwestern.edu

**Table S1**
**Proteins analyzed in this study**

| **Species, enzyme, ( size a.a.)** | **Protein acc. no.** |
|---|---|
| *Arabidopsis thaliana* **DHS** (368) | Q9FI94 |
| *Homo sapiens* **DHS** (369) | AAA96151 |
| *Ca. Fonsibacter ubiquis* **FunubiSpeY** (348) | WP_099339814 |
| *Caldilinea aerophila* **CalaerSpeY** (327) | WP_014432699 |
| *Capsulimonas corticalis* **CapcorSpeY** (321) | WP_119321263 |
| *Ca. Sericytochromatia* bacterium **CaSerSpeY** (343) | MBT9546716 |
| *Rhizorhabdus wittichii* **RhiwitSpeY**(350) | WP_012050836 |
| *Winogradskyella algicola* **WinalgSpeY** (325) | WP_138435141 |
| *Thermus thermophilus* **ThetheSpeY** (340) | WP_164926062 |
| *Magnetospirillum gryphiswaldense* **MaggryHSS** (469) | WP_106001591 |
| *Brucella abortus* **BruaboHSS** (481) | WP_022583674 |
| *Thermus thermophilus* **ThetheSpeB** (298) | WP_011228458 |
| *Anabaena* sp. PCC 7120 **AnabaeSpeB** (346) | WP_010996466 |
| *Winogradskyella algicola* **WinalgSpeB** (313) | WP_138435142 |
| *Caldilinea aerophila* **CalaerSpeB** (301) | WP_014432698 |
| *Escherichia coli* **EcoliSpeB** (306) | BAE77000 |
| *Bacillus subtilis* **BacsubSpeB** (290) | CAB15776 |
| *Magnetospirillum gryphiswaldense* **MaggrySpeB** (295) | WP_106001593 |
| *Ca. Fonsibacter ubiquis* **FunubiAIH** (356) | WP_099339812 |
| *Rhizorhabdus wittichii* **RhiwitAIH** (332) | WP_208633055 |
| *Pseudomonas aeruginosa* **PseaerAIH** (368) | NP_248983 |
| *Enterococcus faecalis* **EntfaeAIH** (365) | EPI20829 |
| *Desulfosporosinus orientis* **DesoriAIH** (345) | WP_014186266 |
| *Rhodothermus marinus* **RhomarAIH** (342) | ACY48398 |
| *Hydrogenimonas thermophila* **HydtheAIH** (340) | WP_317066785 |
| *Brucella abortus* **ODC** (377) | WP_006121644 |
| *Rhodothalassium salexigens* **ODC/ADC** (444) | WP_132707496 |
| *Rhodopila globiformis* **ODC/ADC** (420) | WP_104521400 |

Protein accession numbers are from GenBank. DHS, deoxyhypusine synthase; SpeY, G44G/diagmatine synthase; SpeB, agmatine ureohydrolase homolog; AIH, agmatine deiminase/iminohydrolase (AguA) homolog; ODC, L-ornithine decarboxylase; ADC, L-arginine decarboxylase.

**Table S2**

**High Resolution LC-MS detection of benzoylated homospermidine and G44G**

| **DHS-, SpeY- and HSS-encoding genes in *E. coli*** | **BL21*speD* (AUP)** | **M.E. (ppm)** | **BL21*speG* (AUP)** | **M.E. (ppm)** | **BL21*speB* + arg (AUP)** | **M.E. (ppm)** |
|---|---|---|---|---|---|---|
| **Homospermidine** | | | | | | |
| ***Group 1*** | | | | | | |
| Empty pETDuet-1 | $2.04 \times 10^4$ | -0.5 | $1.51 \times 10^4$ | -5.6 | $1.67 \times 10^4$ | -0.9 |
| *Arabidopsis thaliana* DHS | $3.65 \times 10^4$ | -4.1 | $4.91 \times 10^7$ | -0.7 | $5.09 \times 10^6$ | 0.4 |
| *Homo sapiens* DHS | $7.48 \times 10^4$ | -4.5 | $3.79 \times 10^7$ | 0.6 | $8.35 \times 10^6$ | -0.1 |
| *Ca. Fonsibacter ubiquis* SpeY | $1.04 \times 10^7$ | 0.6 | $4.46 \times 10^6$ | 0.4 | $8.16 \times 10^4$ | -5.3 |
| ***Group 2*** | | | | | | |
| Empty pETDuet-1 | $4.39 \times 10^4$ | -9.9 | $4.92 \times 10^4$ | -6.9 | $4.50 \times 10^4$ | -10.1 |
| *Caldilinea aerophila* SpeY | $1.27 \times 10^8$ | -2.1 | $7.78 \times 10^7$ | 1.2 | $3.96 \times 10^5$ | -5.0 |
| *Capsulimonas corticalis* SpeY | $2.38 \times 10^8$ | 0.8 | $1.48 \times 10^8$ | 1.0 | $8.60 \times 10^5$ | -5.3 |
| *Ca. Sericytochromatia* SpeY | $1.08 \times 10^8$ | 0.7 | $1.37 \times 10^7$ | 0.6 | $8.18 \times 10^5$ | -3.9 |
| *Rhizorhabdus wittichii* SpeY | $7.41 \times 10^7$ | 0.9 | $1.16 \times 10^7$ | 0.6 | $7.88 \times 10^5$ | -7.3 |
| *Winogradskyella algicola* SpeY | $1.20 \times 10^8$ | -2.3 | $9.48 \times 10^7$ | 2.0 | $4.56 \times 10^5$ | -5.8 |
| ***Group 3*** | | | | | | |
| Empty pETDuet-1 | $5.51 \times 10^4$ | -3.3 | $4.01 \times 10^4$ | -6.6 | $1.62 \times 10^5$ | -4.4 |
| *M. gryphiswaldense* HSS | $8.36 \times 10^8$ | 0.6 | $7.20 \times 10^8$ | 2.4 | $1.53 \times 10^7$ | -0.8 |
| *Brucella abortus* HSS | $8.32 \times 10^8$ | 5.2 | $6.72 \times 10^8$ | 3.6 | $3.05 \times 10^7$ | 0.1 |
| **G44G** | | | | | | |
| ***Group 1*** | | | | | | |
| Empty pETDuet-1 | N.D. | -0.9 | $5.28 \times 10^2$ | -4.1 | $1.67 \times 10^4$ | 1.9 |
| *Arabidopsis thaliana* DHS | N.D. | -1.1 | N.D. | -3.3 | $3.69 \times 10^4$ | -3.0 |
| *Homo sapiens* DHS | N.D. | -7.6 | N.D. | -11.8 | N.D. | -7.0 |
| *Ca. Fonsibacter ubiquis* SpeY | $5.54 \times 10^5$ | -2.1 | $8.39 \times 10^5$ | -2.4 | $2.17 \times 10^8$ | -0.8 |
| ***Group 2*** | | | | | | |
| Empty pETDuet-1 | $4.95 \times 10^3$ | -8.4 | $3.52 \times 10^4$ | -8.8 | $2.88 \times 10^4$ | -2.3 |
| *Caldilinea aerophila* SpeY | $1.72 \times 10^5$ | -4.8 | $1.30 \times 10^5$ | -2.3 | $3.11 \times 10^8$ | -0.4 |
| *Capsulimonas corticalis* SpeY | $1.52 \times 10^5$ | -4.1 | $1.14 \times 10^5$ | -4.6 | $3.19 \times 10^8$ | -0.6 |
| *Ca. Sericytochromatia* SpeY | $6.88 \times 10^5$ | -5.2 | $1.26 \times 10^5$ | -4.8 | $2.60 \times 10^8$ | 0.3 |
| *Rhizorhabdus wittichii* SpeY | $5.43 \times 10^6$ | 2.0 | $8.94 \times 10^5$ | -1.8 | $2.31 \times 10^8$ | -0.9 |
| *Winogradskyella algicola* SpeY | $3.35 \times 10^5$ | -4.1 | $1.94 \times 10^5$ | -3.2 | $1.81 \times 10^8$ | -1.4 |
| ***Group 3*** | | | | | | |
| Empty pETDuet-1 | $1.30 \times 10^4$ | -0.4 | $3.92 \times 10^3$ | 3.1 | $1.54 \times 10^4$ | -0.1 |
| *M. gryphiswaldense* HSS | $6.22 \times 10^3$ | -4.6 | $1.29 \times 10^4$ | -1.1 | $6.70 \times 10^3$ | -1.8 |
| *Brucella abortus* HSS | $1.32 \times 10^4$ | 3.5 | $6.68 \times 10^3$ | -1.2 | $1.80 \times 10^4$ | 2.2 |

High Resolution Liquid Chromatography Mass Spectrometry (HR-LCMS) detection of tribenzoylated homospermidine (Hspd) (calculated mass = 472.2594684) and pentabenzoylated G44G (764.355494) in cell extracts of *E. coli* BL21*speD,* BL21*speG* and BL21*speB* grown with 300 µM arginine (arg, Groups 1 & 2) or 2 mM arg (Group 3). M.E. (ppm), mass error (parts per million). *SpeY, dhs and hss* genes are expressed from pETDuet-1. AUP, area under peak; *Ca., Candidatus*; *M. gryphiswaldense*, *Magnetospirillum gryphiswaldense*. All strains from within each group were grown in chemically-defined, polyamine-free M9 medium and analyzed together but each group represents an independent experiment. The *E. coli* BL21*speD*, *speG* and *speB* strains are single gene deletions of *S*-adenosylmethionine decarboxylase, spermidine *N*-acetyltransferase and agmatine ureohydrolase, respectively. N.D., not detected.

**Table S3**
**High Resolution LC-MS detection of benzoylated G44G and homospermidine in cell extracts of *E. coli* BL21*speB*+ 2 mM arginine**

| Expressed genes | AUP 2.78 min (Hspd) | M.E. (ppm) | AUP 3.07 min (G44G) | M.E. (ppm) |
|---|---|---|---|---|
| pETDuet-1 + pACYCDuet-1 | $3.28 \times 10^3$ | -2.5 | $7.77 \times 10^1$ | -2.7 |
| ThetheSpeY + pACYCDuet-1 | $4.09 \times 10^3$ | -1.3 | $2.46 \times 10^6$ | 2.9 |
| FonubiSpeY + pACYCDuet-1 | $7.71 \times 10^3$ | -1.8 | $2.48 \times 10^6$ | -0.2 |
| WinalgSpeY + pACYCDuet-1 | $7.15 \times 10^3$ | -1.1 | $3.41 \times 10^6$ | 1.4 |
| CalaerSpeY + pACYCDuet-1 | $6.76 \times 10^3$ | -1.4 | $4.34 \times 10^6$ | 0.4 |
| RhiwitSpeY + pACYCDuet-1 | $6.00 \times 10^3$ | -1.4 | $3.72 \times 10^6$ | 1.9 |
| CapcorSpeY + pACYCDuet-1 | $6.38 \times 10^3$ | -1.3 | $2.14 \times 10^6$ | 1.3 |
| CaSerSpeY + pACYCDuet-1 | $5.63 \times 10^3$ | -4.1 | $2.98 \times 10^6$ | 0.6 |
| ThetheSpeY + ThetheSpeB | $3.90 \times 10^6$ | 2.5 | $1.02 \times 10^6$ | 1.3 |
| FonubiSpeY + AnabaeSpeB | $7.10 \times 10^6$ | 8.6 | $6.61 \times 10^3$ | -0.7 |
| WinalgSpeY + WinalgSpeB | $6.86 \times 10^6$ | 8.0 | $4.76 \times 10^4$ | -0.1 |
| CalaerSpeY + CalaerSpeB | $4.42 \times 10^6$ | 1.7 | $2.87 \times 10^5$ | 0.7 |
| RhiwitSpeY + AnabaeSpeB | $7.82 \times 10^6$ | 6 | $6.40 \times 10^4$ | 0.7 |
| CapcorSpeY + AnabaeSpeB | $5.50 \times 10^6$ | 6.4 | $2.71 \times 10^4$ | -0.5 |
| CaSerSpeY + AnabaeSpeB | $7.03 \times 10^6$ | 6.4 | $7.79 \times 10^4$ | 0.8 |

High Resolution Liquid Chromatography Mass Spectrometry (HR-LCMS) detection of tribenzoylated homospermidine (Hspd) (m/z = 472.259) and pentabenzoylated G44G (m/z = 764.355) in cell extracts of *E. coli* BL21*speB* grown with 2 mM arginine in M9 medium. SpeY-encoding genes are expressed from pETDuet-1 and SpeB genes from pACYCDuet-1. AUP, area under peak. All strains were grown and analyzed together. Thethe, *Thermus thermophilus*; Fonubi, *Candidatus Fonsibacter ubiquis*; Winalg, *Winogradskyella algicola*; Calaer, *Caldilinea aerophila*; Rhiwit, *Rhizorhabdus wittichii*; Capcor, *Capsulimonas corticalis*; CaSer, *Ca. Sericytochromatia* bacterium; Anabae, *Anabaena* sp. PCC 7120.

**Table S4**
**HR-LCMS detection of homospermidine produced by coexpression of *Capsulimonas corticalis* SpeY- and diverse SpeB-encoding ureohydrolases**

| **Expressed genes in *E. coli* BL21*speB*** | **AUP (Hspd)** | **M.E. (ppm)** |
|---|---|---|
| pETDuet-1 + pACYCDuet-1 | $1.65 \times 10^5$ | -2.8 |
| CapcorSpeY + pACYCDuet-1 | $1.77 \times 10^6$ | -3.3 |
| CapcorSpeY + ThetheSpeB | $5.57 \times 10^8$ | 0.9 |
| CapcorSpeY + AnabaeSpeB | $7.55 \times 10^8$ | 0.8 |
| CapcorSpeY + WinalgSpeB | $7.83 \times 10^8$ | 0.5 |
| CapcorSpeY + CalaerSpeB | $3.82 \times 10^8$ | 1.2 |
| CapcorSpeY + EcoliSpeB | $6.45 \times 10^8$ | 0.3 |
| CapcorSpeY + BacsubSpeB | $4.82 \times 10^8$ | -0.7 |
| CapcorSpeY + MaggrySpeB | $3.57 \times 10^8$ | 1.1 |

HR-LCMS detection of tribenzoylated homospermidine (Hspd) (calculated mass = 472.2594684) in cell extracts of *E. coli* BL21*speB* grown without added arginine. The *speY* gene is expressed from pETDuet-1 and *speB* genes from pACYCDuet-1. AUP, area under peak; All strains were grown and analyzed together. M.E. (ppm), mass error (parts per million); Ecoli, *Escherichia coli*; Bacsub, *Bacillus subtilis*; Maggry, *Magnetospirillum gryphiswaldense.*

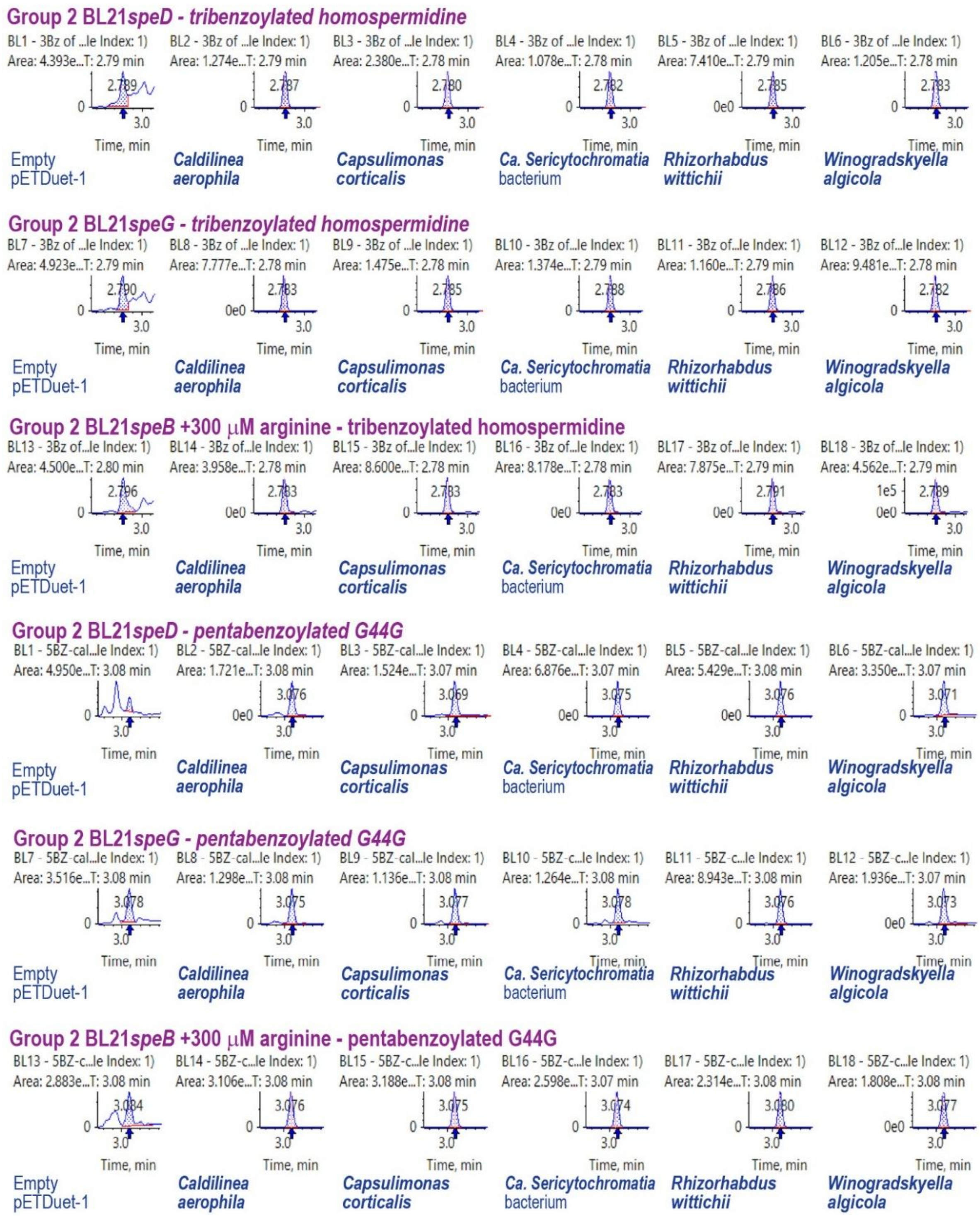


**Figure S1**

HR-LCMS chromatograms of homospermidine and G44G produced by *speY*-expressing *E. coli* BL21 strains relating to Table 1. The chromatograms provide the Area under the Peak (AUP) values for Table 1, and show tribenzoylated homospermidine (m/z 472.259) and pentabenzoylated G44G (m/z 764.355). The y-axes represent arbitrary units of ion intensity.

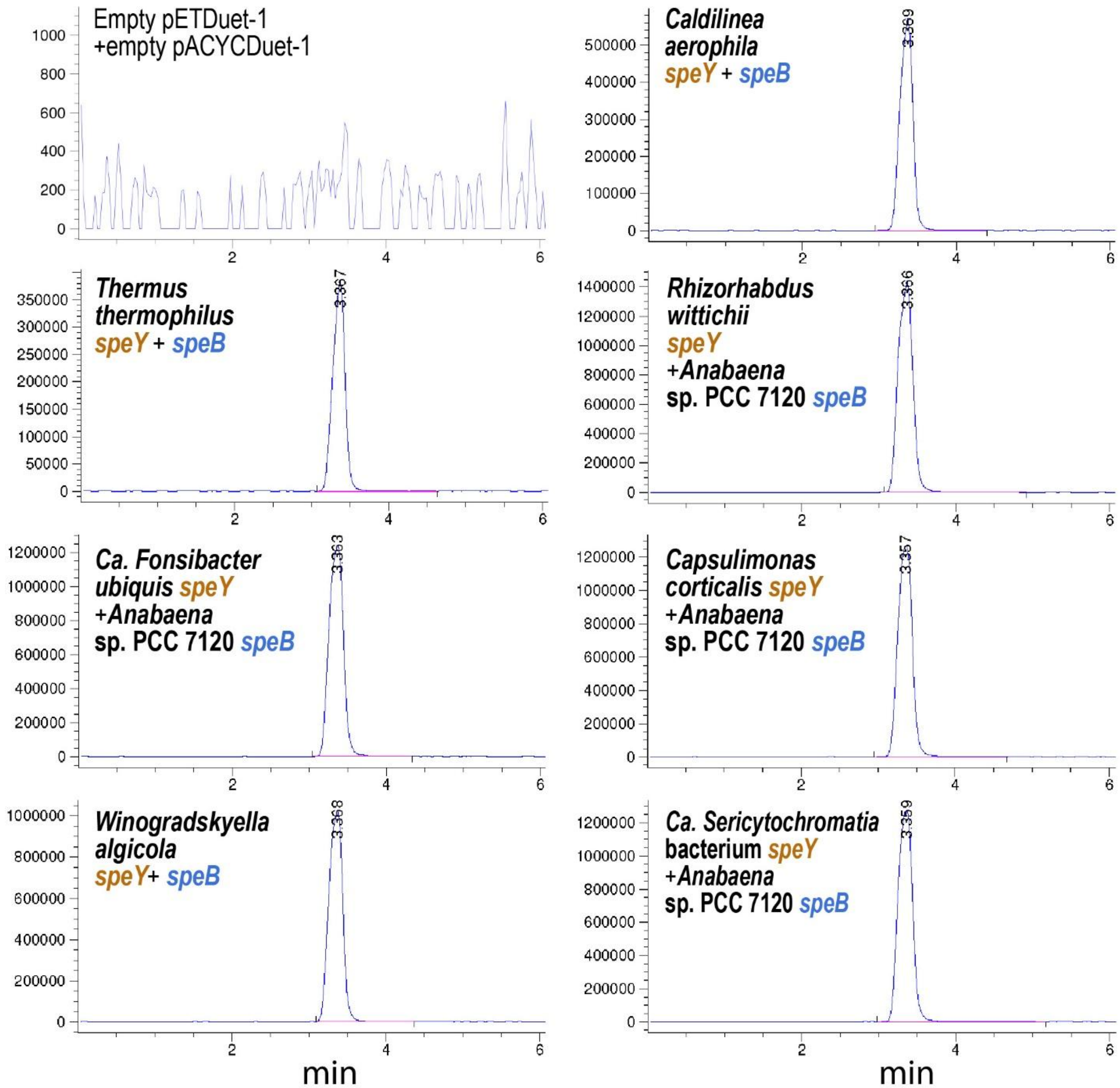


**Figure S2**

LC-MS chromatograms of tribenzoylated homospermidine (mass window m/z 471.9:472.9) produced by *speY*- + *speB*- expressing *E. coli* BL21*speB* strains grown in M9 medium with 2 mM L-arginine, from an independent experiment replicating the experiment shown in Table 1 and Fig. S1. The y-axes represent arbitrary units of ion intensity.

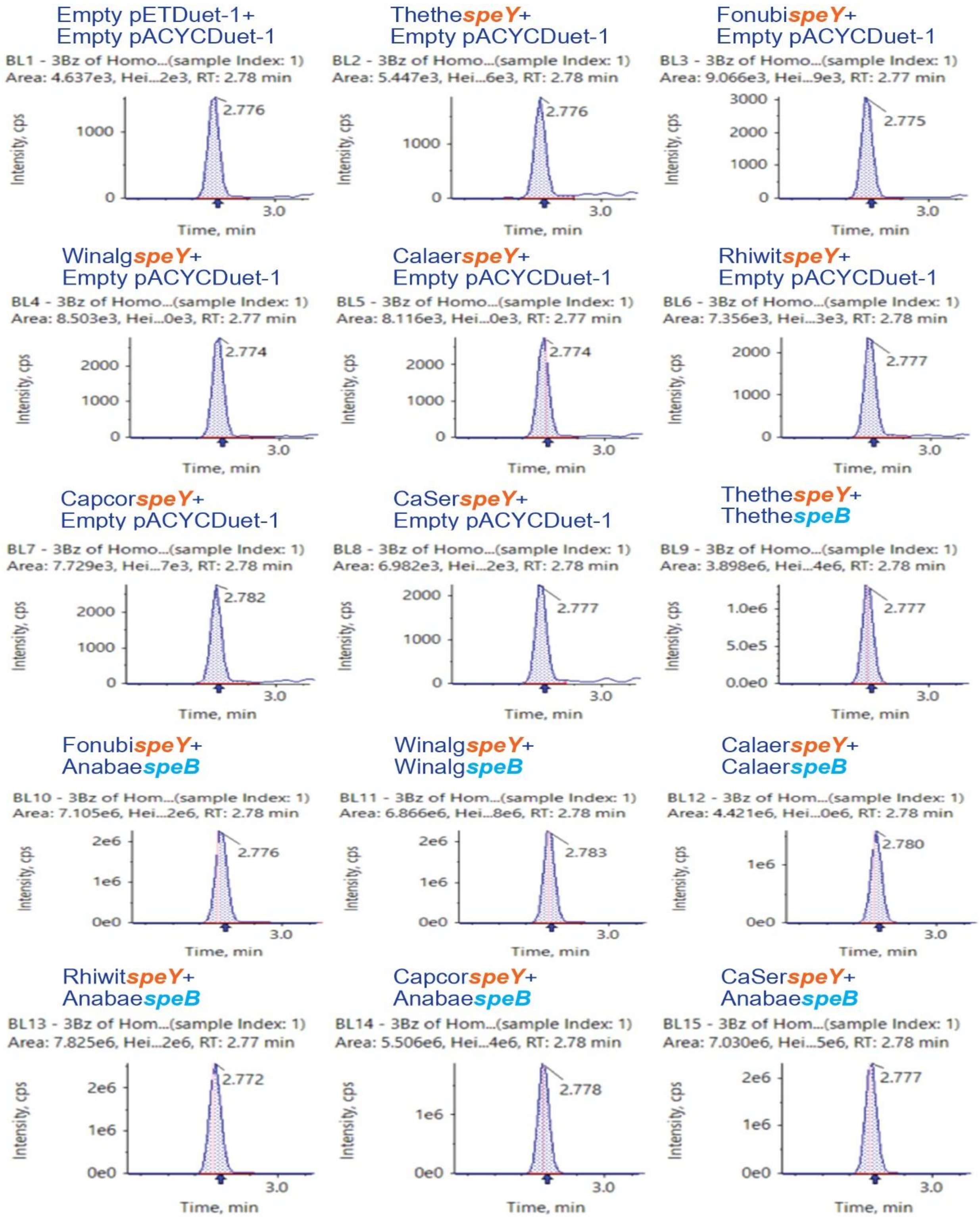


**Figure S3**

HR-LCMS chromatograms for tribenzoylated homospermidine (m/z 472.259) relating to Table 2.

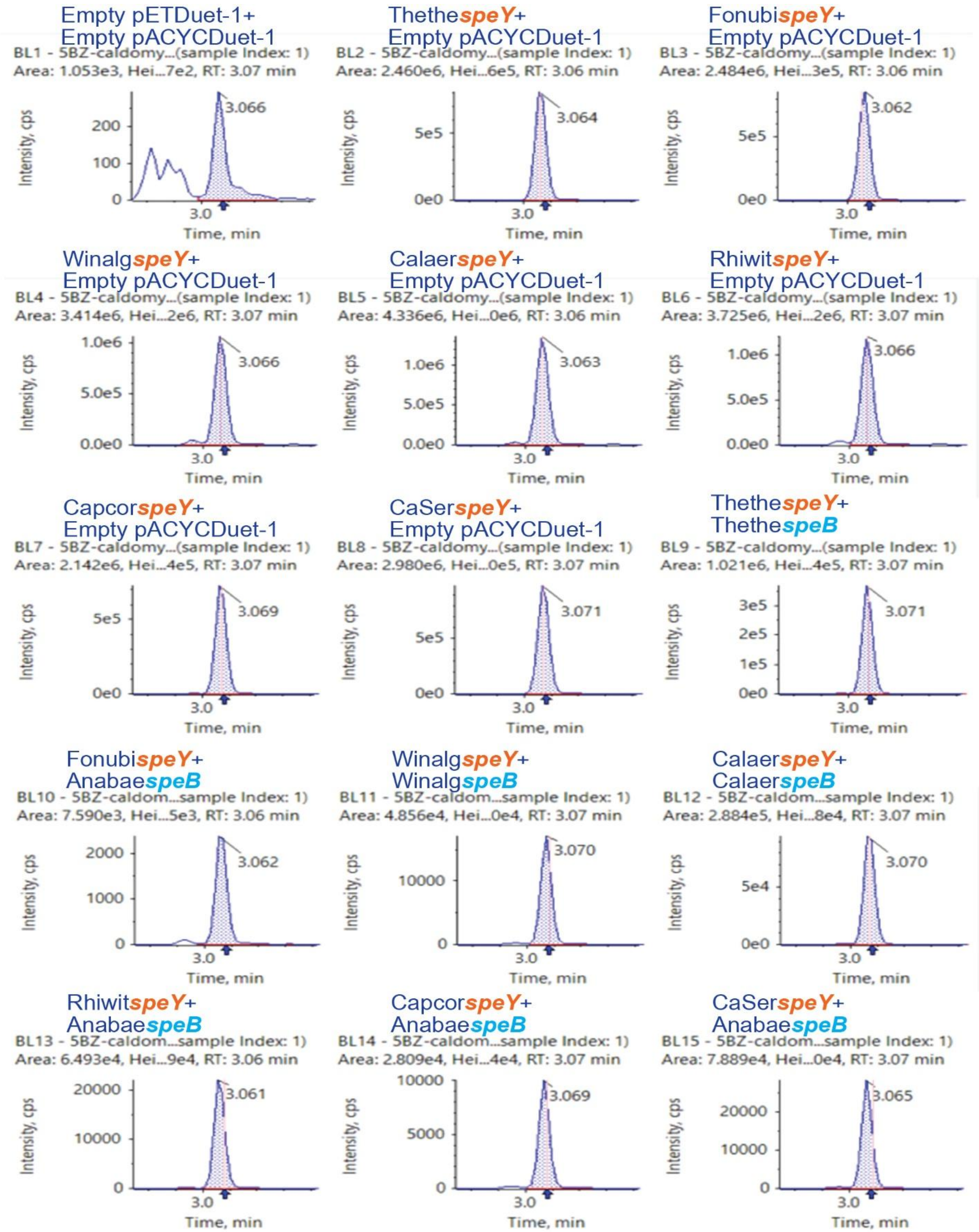


**Figure S4**

HR-LCMS chromatograms for pentabenzoylated G44G (m/z 764.355) relating to Table 2.

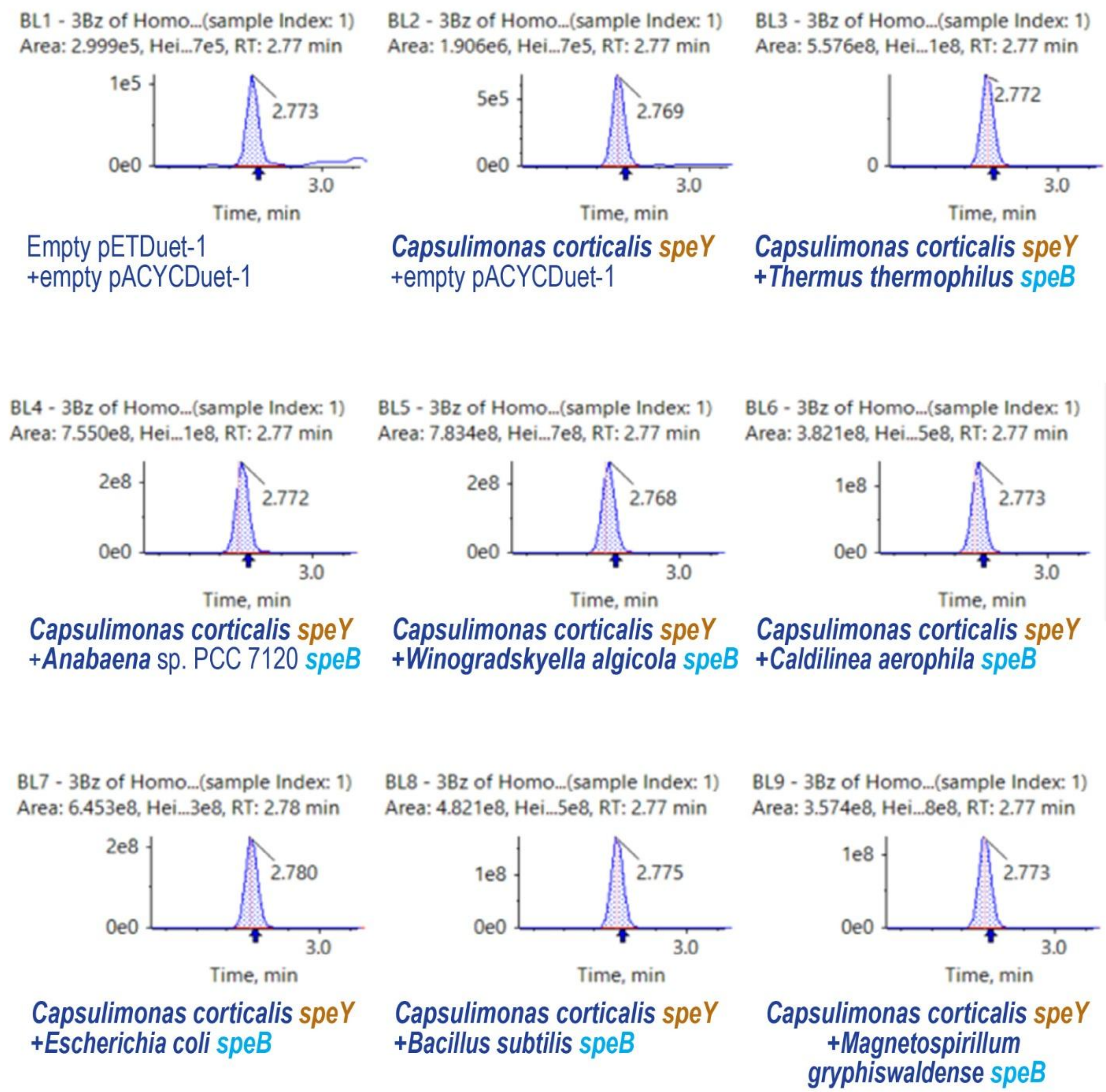


**Figure S5**

HR-LCMS chromatograms for tribenzoylated homospermidine (m/z 472.259) relating to Table 3. The y-axes represent arbitrary units of ion intensity.

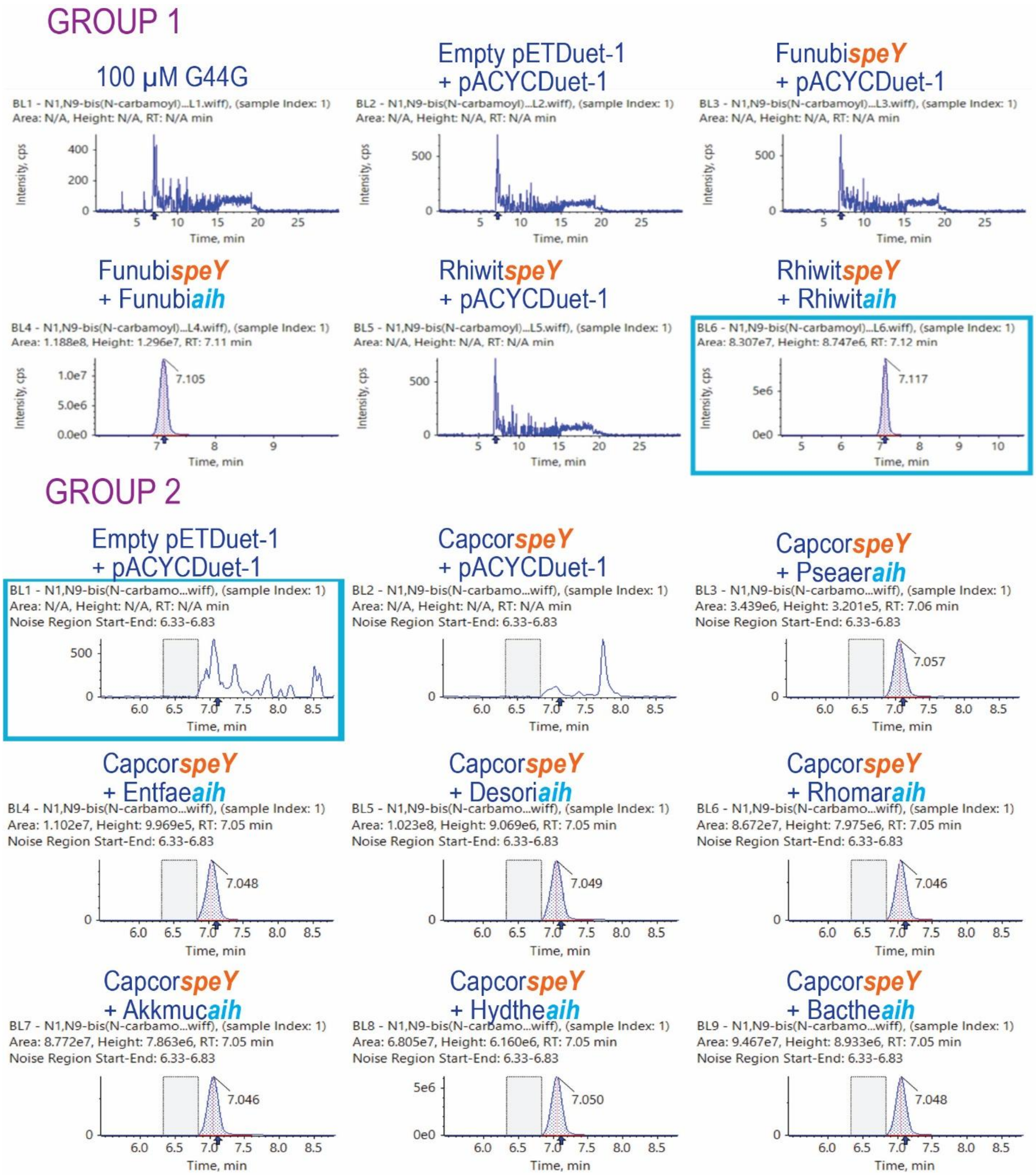


**Figure S6**

High Resolution LC-MS/MS (HR-LCMS) chromatograms of unbenzoylated $N^1$,$N^9$-*bis*(carbamoyl)homospermidine relating to Table 5. The y-axes represent arbitrary units of ion intensity.
